# What explains gender inequalities in HIV/AIDS prevalence in sub-Saharan Africa? Evidence from the demographic and health surveys

**DOI:** 10.1186/s12889-016-3783-5

**Published:** 2016-11-03

**Authors:** Drissa Sia, Yentéma Onadja, Mohammad Hajizadeh, S. Jody Heymann, Timothy F. Brewer, Arijit Nandi

**Affiliations:** 1 Département des sciences infirmières, Campus de Saint-Jérôme, Université du Québec en Outaouais, 5, rue Saint-Joseph, bureau J-3226, Saint Jérôme, Québec J7Z 0B7 Canada; 2 Institut Supérieur des Sciences de la Population (ISSP), Université Ouaga I Pr Joseph Ki-Zerbo, 03 BP 7118 Ouagadougou 03, Ouagadougou, Burkina Faso; 3 School of Health Administration, Dalhousie University, Halifax, Nova Scotia Canada; 4 Fielding School of Public Health, The University of California, Los Angeles (UCLA), Los Angeles, CA USA; 5 David Geffen School of Medicine, The University of California, Los Angeles (UCLA), Los Angeles, CA USA; 6 Institute for Health and Social Policy and Department of Epidemiology, Biostatistics, and Occupational Health, McGill University, Montreal, Québec Canada

**Keywords:** Gender inequality, HIV/AIDS, Blinder-Oaxaca decomposition, Sub-Saharan Africa

## Abstract

**Background:**

Women are disproportionally affected by human immunodeficiency virus (HIV)/acquired immunodeficiency syndrome (AIDS) in sub-Saharan Africa (SSA). The determinants of gender inequality in HIV/AIDS may vary across countries and require country-specific interventions to address them. This study aimed to identify the socio-demographic and behavioral characteristics underlying gender inequalities in HIV/AIDS in 21 SSA countries.

**Methods:**

We applied an extension of the Blinder-Oaxaca decomposition approach to data from Demographic and Health Surveys and AIDS Indicator Surveys to quantify the differences in HIV/AIDS prevalence between women and men attributable to socio-demographic factors, sexual behaviours, and awareness of HIV/AIDS. We decomposed gender inequalities into two components: the percentage attributable to different levels of the risk factors between women and men (the “composition effect”) and the percentage attributable to risk factors having differential effects on HIV/AIDS prevalence in women and men (the “response effect”).

**Results:**

Descriptive analyses showed that the difference between women and men in HIV/AIDS prevalence varied from a low of 0.68 % (*P* = 0.008) in Liberia to a high of 11.5 % (*P* < 0.001) in Swaziland. The decomposition analysis showed that 84 % (*P* < 0.001) and 92 % (*P* < 0.001) of the higher prevalence of HIV/AIDS among women in Uganda and Ghana, respectively, was explained by the different distributions of HIV/AIDS risk factors, particularly age at first sex between women and men. In the majority of countries, however, observed gender inequalities in HIV/AIDS were chiefly explained by differences in the responses to risk factors; the differential effects of age, marital status and occupation on prevalence of HIV/AIDS for women and men were among the significant contributors to this component. In Cameroon, Guinea, Malawi and Swaziland, a combination of the composition and response effects explained gender inequalities in HIV/AIDS prevalence.

**Conclusions:**

The factors that explain gender inequality in HIV/AIDS in SSA vary by country, suggesting that country-specific interventions are needed. Unmeasured factors also contributed substantially to the difference in HIV/AIDS prevalence between women and men, highlighting the need for further study.

**Electronic supplementary material:**

The online version of this article (doi:10.1186/s12889-016-3783-5) contains supplementary material, which is available to authorized users.

## Background

Countries in sub-Saharan Africa (SSA) remain the most severely affected by the human immunodeficiency virus (HIV)/acquired immunodeficiency syndrome (AIDS) pandemic, accounting for 68 % of all persons living with HIV/AIDS worldwide [1, 2]. Compared to men, women living in SSA are disproportionally affected by HIV/AIDS, accounting for 59 % of all infections in this region [1–3]. The gender disparity starts at a young age, with 15 to 24 year old women in SSA being more than twice as likely as men to become newly infected with HIV [1, 2, 4]. There is an increasing recognition that prevention and treatment programs must address gender inequalities in HIV/AIDS [5]. Nevertheless, the mechanisms that give rise to these inequalities are poorly understood.

Gender inequalities in HIV/AIDS might be attributable to the differential distribution of risk factors for women and men. For example, the lower socioeconomic position of women in SSA may place them at greater behavioural risk for HIV infection [6–8]. Women are more likely to be uneducated, unemployed, and impoverished than men, which predisposes them to transactional sexual exchanges [9]. These sexual exchanges are often made with casual sex partners and without protection [10]. Thus, economic inequality between women and men may increase vulnerability to HIV among sexually active women [11]. Besides economic differences, unequal power relationships and the subordinate position of women relative to men also place women at higher risk for contracting HIV [12–18]. Women in SSA generally have less power to negotiate safe sex, including condom use [19]. Additionally, cultural factors encouraging older men—who are more likely to be HIV-infected—to have younger female partners (i.e., intergenerational sex) [20] limit women’s ability to negotiate safe sex and increase the risk of HIV infection for women relative to men potentially exacerbating gender inequalities in HIV infection [21]. Moreover, social norms permitting violence against women, including domestic violence, spousal abuse, and rape might increase the probability of infection among women. This violence has many implications for the spread of HIV/AIDS. For example, it is associated with lack of condom use as well as traumatic injury among women in SSA [19], which increases risk of HIV infection [16, 22–25].

The differential responses of women and men to HIV/AIDS risk factors may also contribute to observed gender inequalities in HIV/AIDS. For example, a recent study by Magadi [3] using pooled data from 20 SSA countries showed that conditioning on HIV risk factors, including sexual behaviors, did not explain the increased odds of HIV/AIDS among women relative to men [3], suggesting that traditional HIV risk factors may have differential and more detrimental effects for women compared to men. Few studies [26, 27], however, have assessed whether risk factors have different effects on the probability of HIV/AIDS for men and women. One study showed that unmarried women have twice the risk of HIV compared to unmarried men [28], suggesting that the differential effects of marital status may contribute to gender inequalities in HIV/AIDS. Additionally, although men and women may have similar distributions of household wealth, women have less control over household decision-making and financial resources and thereby may lack power to negotiate safe sexual practices (for example, condom use) with their partners [9, 29, 30], which puts them at higher risk for HIV/AIDS. Unmeasured biological factors may also be important [31]. For example, male-to-female transmission of HIV is more biologically efficient than female-to-male transmission [32–35]. However, gender inequalities in HIV/AIDS vary substantially across world regions and are unlikely to be explained by biological differences alone. Political, organizational and legislative [5], social [12–18], and other cultural factors not already mentioned may also play important roles.

Gender inequalities in HIV/AIDS prevalence vary across countries [36]. Clarifying the determinants of gender inequalities in the SSA region, including whether they are explained by the differential distributions (a “*composition effect*”) or effects (a “*response effect*”) of HIV/AIDS risk factors for women and men, may help to inform country-specific interventions for mitigating them [37, 38]. However, the characteristics explaining gender inequalities in HIV/AIDS prevalence in this region have not been systematically evaluated. Using data from the Demographic and Health Surveys (DHS) and AIDS indicator surveys (AIS), we recently elucidated the factors explaining gender inequalities in HIV/AIDS prevalence in Kenya, Lesotho and Tanzania [39]. This study showed that composition effects mainly explained gender inequalities in HIV/AIDS in Tanzania, whereas in Kenya and Lesotho they were partly explained by differences in the effects of measured HIV/AIDS risk factors for men and women, including socio-demographic characteristics (i.e., age and marital status) and sexual behaviours (i.e., age at first sex). In the current study, we extended our previous work by: 1) measuring the magnitude of the gender inequality in HIV/AIDS prevalence across 21 SSA countries using available DHS; 2) quantifying the extent to which gender inequalities in HIV/AIDS were attributable to composition or response effects using a decomposition analysis; and 3) estimating the contribution of each risk factor to gender inequalities in HIV/AIDS prevalence across SSA countries.

## Methods

### Data

We used available data from the international DHS and the AIS to analyse the sources of gender inequality in HIV/AIDS prevalence across 21 SSA countries surveyed between 2003 and 2012 (Table 1). Each DHS is a cross-sectional survey that collects and disseminates nationally representative household data, including comparable information on socio-demographic, behavioral, nutritional, health and other characteristics over time [22, 40, 41]. The DHS uses a multistage stratified design with probabilistic sampling that gives a defined probability of selection to each elementary unit [42]. Each DHS survey was stratified by urban and rural status and also by country-specific geographic or administrative regions [43]. To ensure comparability across countries and time, the DHS uses standardized measurement tools and techniques and an identical core questionnaire that is pretested and then administered by trained interviewers [44]. Further details concerning the DHS survey methodology are available elsewhere [45].

**Table 1:** Response rates (%), samples size and prevalence of HIV/AIDS (%) by gender, country and survey year

	Survey year	Age range (years)		Response rates (%)^a^	Women			Men			Gender inequality in HIV/AIDS prevalence	
Countries		Women	Men		n^b^	HIV +^c^	Prevalence1 (%)^d^	n^b^	HIV +^c^	Prevalence (%)^d^	Women-Men	*p*-value^e^
Burkina Faso (BF)	2003	15–49	15–59	89	4189	84	1.82	3341	59	1.95	−0.13	0.713
	2010	15–49	15–59	95	8346	100	1.18	7034	60	0.84	0.34	0.086
Cameroon (CM)	2004	15–49	15–59	91.34	5154	349	6.63	5041	203	3.92	2.71	<0.001
	2011	15–49	15–59	93	7253	434	5.57	6945	215	2.89	2.69	<0.001
Congo Brazzaville (CG)	2009	15–49	15–49	97.6	6349	240	4.13	5760	134	2.06	2.07	<0.001
Côte d’Ivoire (CI)	2005	15–49	15–49	78	4547	247	6.21	3917	110	3.11	3.1	<0.001
Ethiopia (ET)	2005	15–49	15–59	79	5942	142	1.86	5107	70	0.91	0.95	0.003
	2011	15–49	15–59	85.75	15505	358	1.86	12998	182	0.98	0.88	<0.001
Ghana (GH)	2003	15–49	15–59	85	5289	138	2.71	4265	68	1.63	1.08	<0.001
Guinea (GN)	2005	15–49	15–59	91	3842	68	1.89	2925	35	1.1	0.79	0.010
Liberia (LR)	2005	15–49	15–49	84	6482	147	1.91	5206	62	1.23	0.68	0.008
Malawi (MW)	2004	15–49	15–54	67	2864	421	13.32	2404	243	10.23	3.09	0.002
	2010	15–49	15–54	87	7396	890	12.88	6509	530	8.39	4.5	<0.001
Mali (ML)	2006	15–49	15–59	88	4743	69	1.54	3886	38	1.11	0.44	0.109
Mozambique (MZ)	2009	15–64	15–64	91	5901	875	12.67	4404	442	9.04	3.63	<0.001
Niger (NE)	2006	15–49	15–59	88	4441	39	0.71	3232	33	0.71	0	0.974
D.R. Congo (CD)	2007	15–49	15–59	88	4632	81	1.62	4304	43	0.92	0.7	0.027
Rwanda (RW)	2005	15–49	15–59	96.5	5663	222	3.61	4728	115	2.2	1.41	<0.001
	2010	15–49	15–59	98	6952	266	3.71	6296	154	2.41	1.3	<0.001
Sao Tome & Principe (ST)	2008/2009	15–49	15–59	--	2550	37	1.29	2160	39	1.8	−0.5	0.215
Senegal (SN)	2005	15–49	15–59	80	4466	48	0.88	3250	16	0.44	0.44	0.009
	2011	15–49	15–59	80	5590	61	0.83	4327	32	0.51	0.32	0.071
Sierra Leone (SL)	2008	15–49	15–59	86	3466	64	1.73	3009	32	1.16	0.57	0.068
Swaziland (SZ)	2006/2007	15–49	15–49	85	4584	1438	31.15	3602	704	19.7	11.45	<0.001
Uganda (UG)	2011	15–59	15–59	96	11967	944	8.21	9399	551	6.11	2.1	<0.001
Zambia (ZM)	2007	15–49	15–59	75	5713	947	16.09	5161	649	12.29	3.8	<0.001
Zimbabwe (ZW)	2005/2006	15–49	15–54	70	7494	1553	21.12	5555	782	14.75	6.37	<0.001
	2010/2011	15–49	15–54	75	7852	1463	17.71	6045	811	12.45	5.05	<0.001

*Note*: *n* sample size

^a^More information on response rates is available at the following link: http://www.measuredhs.com/What-We-Do/survey-search.cfm?pgtype=main&SrvyTp=country

^b^Numbers of women and men in the sample. These frequencies are unweighted numbers

^c^Numbers of women and men with HIV positive test. These frequencies are unweighted numbers

^d^A Weighted percentage of persons with HIV positive test among women and men using sampling weights provided by the DHS and AIS

^e^ *p*-value based on Chi-squared test for the difference in HIV/AIDS prevalence between women and men

The AIS has been fielded in selected low- and middle-income countries since 2001 [3, 46]. Unlike sentinel surveillance, the AIS is a population-based survey that provides nationally-representative HIV prevalence data based on anonymous and voluntary testing of men and women aged 15–49 who were interviewed in the DHS, although some countries have also tested older adults [47, 48]. Due to the anonymous nature of the survey, respondents cannot be provided with their results. However, all respondents are offered referrals for free voluntary counselling and testing (VCT) and AIDS educational materials. In some countries, mobile VCT teams follow up after interviewers to counsel and test respondents who agree to be tested. The comparative nature of the DHS and the possibility to link HIV status from the AIS to the full DHS survey data, while conserving anonymity, provide a unique opportunity to examine factors contributing to gender inequalities in HIV/AIDS in different contexts in Africa. Data from the standard DHS linked to HIV prevalence data from the AIS were available for 313,207 respondents across 21 countries, with seven countries surveyed twice between 2003 and 2012.

We used secondary data collected by the international Demographic and Health Survey (DHS) program after obtaining participants’ consent. Due to the anonymous nature of our data, our study was exempted from ethical ​committee review.

### Measures

Our outcome of interest was HIV serostatus, determined by a confirmatory HIV-positive antibody blood serum result. Sex of the respondent (male vs. female, as defined in the DHS and AIS), used as a proxy for gender, was the key explanatory variable. Other covariates included socio-demographic characteristics, sexual behaviours, and HIV/AIDS awareness. Socio-demographic characteristics included urban/rural residence, the sex of the household head, the respondent’s age at the time of survey, educational attainment (none, primary, or secondary and above), marital status (married, never married, or separated/divorced/widowed), and occupational type (agricultural, unemployed, domestic, trade, manual, office/service, or professional/manager). Applying a relative approach to poverty [49–51], household wealth was measured by a composite index created by principal component analysis (PCA) using information on household assets (ownership of radio, television, refrigerator, bicycle, motorcycle/scooter, car/truck, and telephone), housing quality, and environmental conditions (electricity, source of drinking water, type of toilet facility, floor material). The wealth index was split into country-specific quintiles. Sexual behaviors included age at first marriage, age at first sex, premarital sex, sexual behavior risk (i.e., if a condom was not used at last sexual intercourse or having intercourse with a partner other than a spouse), and having multiple sex partners in the past year. Following the approach of Magadi [3], the PCA technique was employed to create a country-specific index of HIV/AIDS awareness using nine questions on knowledge of the modes of HIV transmission and ways to avoid infection.

### Statistical analysis

We calculated the prevalence of HIV/AIDS for women and men across countries. The Chi-square test was used to estimate gender inequalities in HIV/AIDS as the difference in prevalence comparing women to men. Then, in countries where gender was significantly associated with HIV/AIDS prevalence, we explored the sources of gender inequalities in HIV/AIDS prevalence using an extension of the Blinder-Oaxaca (BO) decomposition [52, 53]. This involved decomposing the observed women-men gaps in the prevalence of HIV/AIDS into two components: composition and response effects. Composition effects represent the contribution to gender inequalities in HIV/AIDS prevalence due to gender differences in the distributions of observable HIV/AIDS risk factors between women and men (i.e., socio-demographic characteristics, sexual behaviors, and HIV/AIDS awareness). Response effects reflect the contribution to gender inequalities in HIV/AIDS due to gender differences in the effects of measured HIV/AIDS risk factors, as well as unmeasured factors not included in the model [52–54]. The percentage of gender inequality in HIV/AIDS explained by a given component for each risk factor is defined by the amount of the difference in HIV/AIDS prevalence explained by the component divided by the total difference in HIV/AIDS prevalence between women and men multiplied by 100. The BO method allowed us to assess which factors were associated with each source of inequality. Initially limited to continuous dependent variables, the BO decomposition approach has been extended to the case of non-linear dependent variables [55–59]. Estimates were obtained using the statistical routine designed for non-linear outcomes described by Powers, Yoshioka and Yun [54]. This approach overcomes potential problems related to path dependence and identification [54]. All analyses, both descriptive and multivariate, were weighted using the available DHS sampling weights and accounted for clustering at the household level. We used STATA version 12 software for all analyses.

## Results

### Gender inequalities in HIV/AIDS

**Fig. 1:**
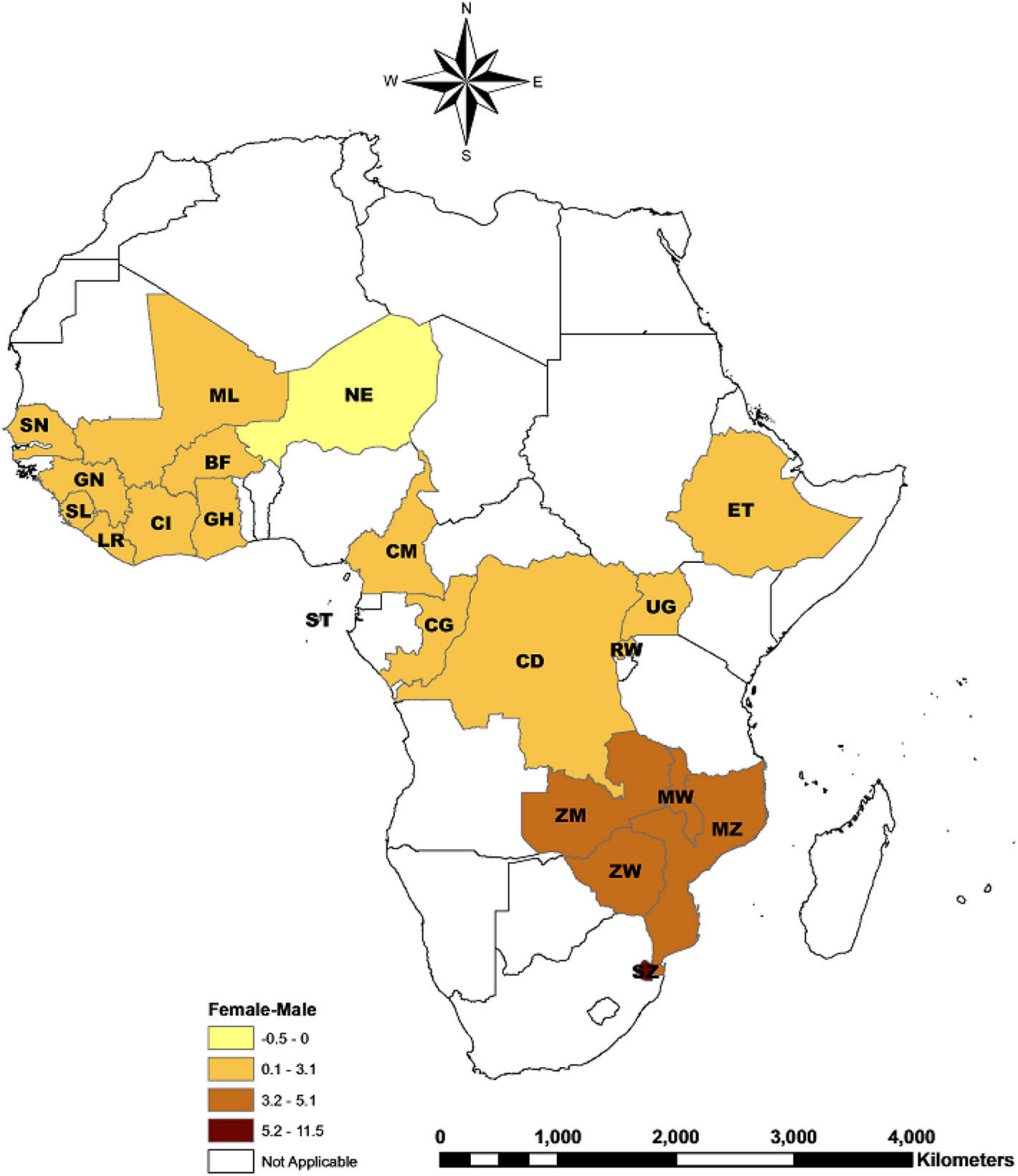
Gender inequalities in HIV/AIDS prevalence in SSA countries

Table 1 reports response rates, samples size and prevalence of HIV/AIDS by gender, country and survey year. Women had a significantly higher prevalence of HIV/AIDS than men in all countries and years sampled, apart from Burkina Faso in 2003 and 2010, Mali in 2006, Niger in 2006, Sao Tome & Principe in 2008/09, Senegal in 2011 and Sierra Leone in 2011. The absolute difference between women and men in HIV/AIDS prevalence ranged from a low of 0.68 % (*P* = 0.008) in Liberia (2005) to a high of 11.5 % (*P* < 0.001) in Swaziland (2006–7). Fig. 1 maps gender inequalities in HIV/AIDS prevalence in 21 SSA countries (using the most recent survey for countries with more than one available); inequalities were more pronounced in the southeastern region of SSA relative to the northwestern region.

### Sample characteristics

Descriptive analyses (Additional file 1: Table S1) showed that there were differences in the distributions of HIV/AIDS risk factors between women and men. On average, women were younger than men in all countries other than Mozambique, Swaziland, Liberia, Zimbabwe and Malawi, likely due to differences in the sampling frames for women and men, which ranged from 15 to 49 years for women and 15 to 64 years for men. In general, compared to men, women were more likely to be married (e.g., 76.7 % versus 63.8 % in Sierra Leone; 62.8 % versus 50 % in Cameroon) and to be separated/divorced/widowed (e.g., 9.1 % versus 5.4 % in Ghana; 18.4 % versus 5.5 % in Mozambique). However, there were some exceptions. For example, in Malawi the percentages of married women and married men were statistically similar. There was no difference between the percentages of separated/divorced/widowed women and men in Mali. In general, women were more socioeconomically disadvantaged than men. For example, compared to men, fewer women had secondary or above education (e.g., 11.1 % versus 31.9 % in Guinea; 25.3 % versus 36.6 % in Uganda). Additionally, women were more likely than men to be unemployed or employed in trading, whereas men were more likely to be employed in professional/managerial occupations. The descriptive statistics results also showed that a higher percentage of women reported first sexual intercourse before 16 years compared to men (e.g., 49.4 % versus 19.4 % in Guinea; 42.3 % versus 27.8 % in Côte d’Ivoire). With the exception of Swaziland, premarital sex was less common among women compared to men (e.g., 32.2 % versus 65.9 % in Malawi; 17.1 % versus 41.8 % in Rwanda). Women had lower levels of HIV/AIDS awareness compared to men, although this was not the case in all countries, for example in Swaziland.

### Explaining gender inequalities in HIV/AIDS prevalence

**Fig. 2:**
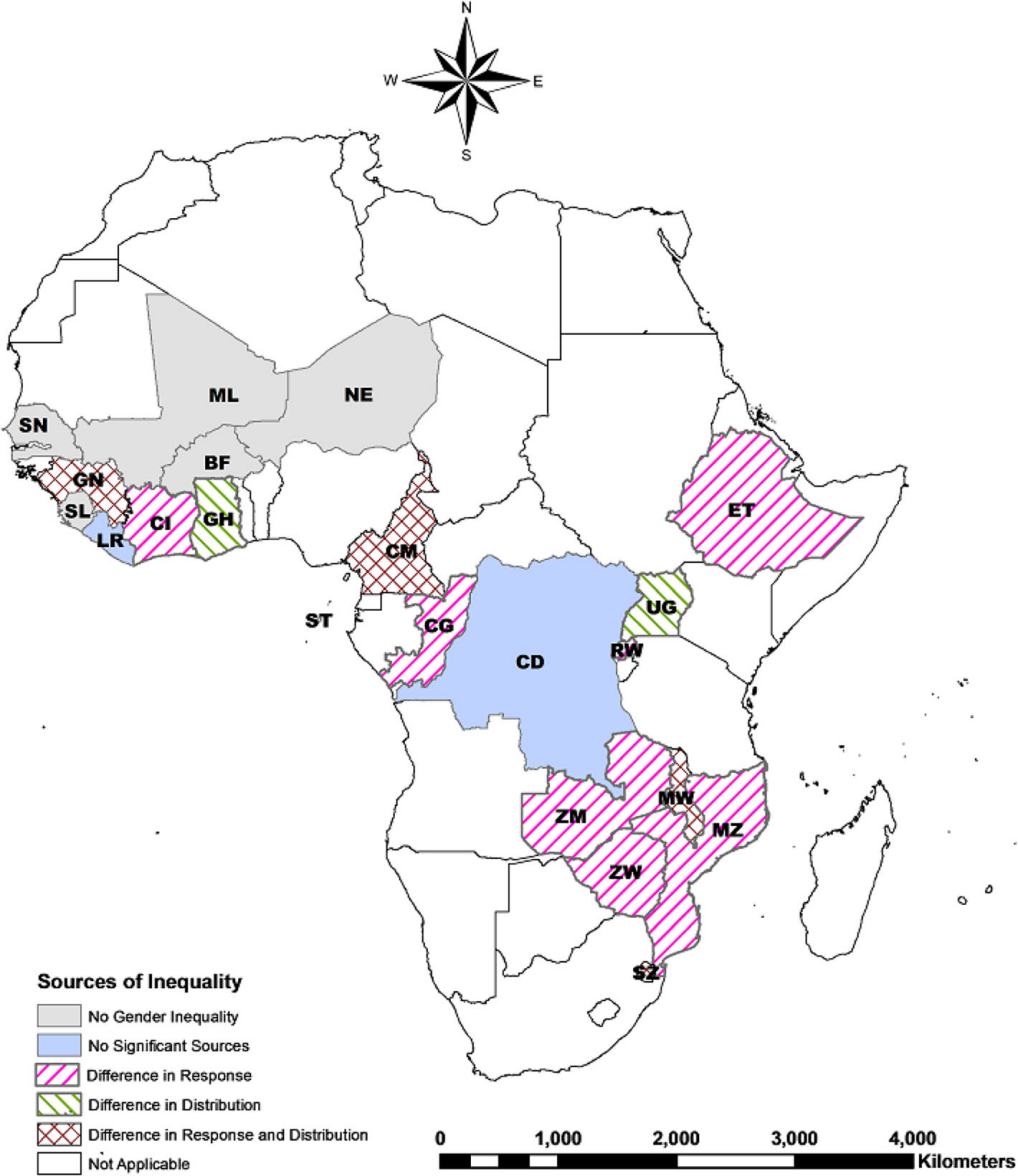
Sources of gender inequalities in HIV/AIDS prevalence among SSA countries

We used the BO decomposition technique to examine sources of gender inequality in HIV/AIDS prevalence across countries (Table 2). There were three distinct patterns (Fig. 2). First, in the majority of countries, the response effect (the differential effect of a risk factor on women and men) explained the concentration of HIV/AIDS among women; the percentage of the gender inequality in HIV/AIDS attributable to this component ranged from 81.5 % in Mozambique and Rwanda to 116 % in Congo Brazzaville. These results indicate that, had responses to HIV/AIDS risk factors been equivalent for men and women, the prevalence of HIV/AIDS would have been 19 % lower among men relative to women in Mozambique and Rwanda and 16 % higher among men relative to women in Congo Brazzaville. Second, in Uganda and Ghana, the composition effect (i.e., differen﻿ce in distribution﻿; ﻿the differential distribution of risk factors by gender) explained 84 % and 92 % of the higher prevalence of HIV/AIDS for women compared to men, respectively. Third, in Cameroon, Guinea, Malawi and Swaziland, both response and composition effects explained gender inequalities in HIV/AIDS prevalence. More than one-half of the gender inequality in HIV/AIDS prevalence in these countries was attributable to gender differences in responses to socio-demographic characteristics, sexual behaviors, HIV⁄AIDS awareness, and unmeasured risk factors.

**Table 2:** Results from Blinder-Oaxaca decomposition analysis of gender inequalities in HIV/AIDS prevalence

Countries	Survey year	Gender inequality in HIV/AIDS prevalence (women-men)	Composition effect^f^			Response effect^g^		
			Beta (SE)	*p*-value	Percent^d^	Beta (SE)	*p*-value	Percent^e^
Cameroon^c^	2011	2.69	0.012 (0.004)	0.001	44.2	0.015 (0.005)	0.002	55.8
Congo Brazzaville^a^	2009	2.07	−0.003 (0.005)	0.478	−15.6	0.024 (0.007)	0.000	115.6
Côte d’Ivoire^a^	2005	3.10	0.005 (0.007)	0.514	16.2	0.024 (0.010)	0.013	83.8
Ethiopia^a^	2011	0.88	0.001 (0.003)	0.698	13.3	0.007 (0.004)	0.039	86.7
Ghana^b^	2003	1.08	0.01 (0.003)	0.000	91.9	0.001 (0.004)	0.811	8.1
Guinea^c^	2005	0.79	−0.014 (0.007)	0.05	−176.5	0.023 (0.008)	0.006	276.5
Liberia	2007	0.68	−0.008 (0.008)	0.314	−111.1	0.014 (0.009)	0.094	211.1
Malawi^c^	2010	4.50	0.022 (0.006)	0.000	48.8	0.023 (0.008)	0.006	51.2
Mozambique^a^	2009	3.63	0.006 (0.011)	0.583	18.5	0.026 (0.013)	0.051	81.5
D.R. Congo	2007	0.70	−0.001 (0.004)	0.852	−10.6	0.008 (0.005)	0.106	110.6
Rwanda^a^	2010	1.30	0.002 (0.004)	0.524	18.6	0.01 (0.005)	0.028	81.4
Swaziland^c^	2006/07	11.45	0.021 (0.009)	0.015	18.7	0.093 (0.012)	0.000	81.3
Uganda^b^	2011	2.10	0.018 (0.003)	0.000	83.7	0.003 (0.005)	0.476	16.3
Zambia^a^	2007	3.80	0.005 (0.008)	0.522	13.9	0.031 (0.01)	0.003	86.1
Zimbabwe^a^	2010/11	5.05	0.009 (0.007)	0.188	17.7	0.044 (0.010)	0.000	82.3

*Note*: using this method, the net percent contribution of both components always equals to 100. A contribution may be negative (less than zero) or positive and can even exceed 100. A positive contribution indicates that the component contributes to the greater prevalence of HIV/AIDS among women relative to men, whereas a negative contribution indicates the opposite

*SE:* Standard Error

^a^Countries where the difference between men and women in the response to risk factors mainly explains the gender gap at *p*-value = 5 %

^b^Countries where the difference in the distribution of risk factors between men and women mainly explains the gender gap at *p*-value = 5 %

^c^Countries where difference in both the response to factors and the distribution of factors between men and women explains the gender gap at *p-value * = 5 %

^d^Part of gender inequality in HIV/AIDS prevalence attributable to differences in the distribution of risk factors

^e^Part of gender inequality in HIV/AIDS prevalence attributable to differences in the effects of risk factors

^f^Represent the contribution to gender inequalities in HIV/AIDS prevalence due to gender differences in the distributions of observable HIV/AIDS risk factors between women and men

^g^Reflect the contribution to gender inequalities in HIV/AIDS due to gender differences in the effects of measured HIV/AIDS risk factors, as well as unmeasured factors not included in the model

**Fig. 3:**
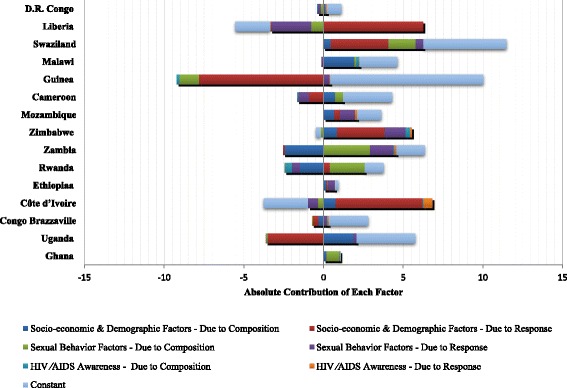
Contribution of factors to gender inequalities in HIV/AIDS prevalence by country

Table 3 and Fig. 3 report detailed results of the decomposition analysis conducted within each country. Gender inequalities in the majority of countries were explained by the differential effects of socio-demographic factors, including age, marital status, and occupation, on prevalence of HIV/AIDS for women and men. Women aged 20–39 were more likely to be HIV-infected than men of the same age group, particularly in Congo Brazzaville (2009), Zambia (2007), and Zimbabwe (2010–11). The increased prevalence of HIV/AIDS among unmarried women compared to unmarried men explained 16.1 % of the gender inequality in Côte d’Ivoire (2005), 43.1 % in Rwanda (2010), 67.4 % in Zambia (2007), and 75 % in Zimbabwe (2010–11). Additionally, the differential effects of occupational status, particularly work in agriculture, contributed to higher HIV/AIDS prevalence for women compared to men in Côte d’Ivoire (68.1 %), Ethiopia (50 %), Mozambique (43.8 %), and Rwanda (23.8 %). The constant term, which comprises the differential effects of factors not included in the model, was the largest contributor to gender inequalities in Congo Brazzaville.

**Table 3:** Contribution of HIV/AIDS risk factors to gender inequalities in HIV/AIDS prevalence by country; decomposition analysis using latest available DHS, 2003 to 2011

	Ghana^a^ 2003	Uganda 2011	Congo Brazzaville^b^ 2009	Côte d’Ivoire^a^ 2005	Ethiopia^a^ 2011	Rwanda 2010	Zambia^a^ 2007	Zimbabwe 2010/11	Mozambique^c^ 2009	Cameroon 2011	Guinea^a^ 2005	Malawi 2010	Swaziland^a^ 2006/07	Liberia 2007	D.R. Congo^d^ 2007
	Absolute contribution^e^ to gender inequalities in HIV/AIDS prevalence (*p*-value)														
Gender Difference in HIV/AIDS Prevalence (%)	1.08	2.1	2.07	3.1	0.88	1.3	3.8	5.05	3.63	2.69	0.79	4.5	11.45	0.68	0.7
*The composition effect* ^*hg*^	0.99 (0.000)	1.76 (0.000)	−0.32 (0.478)	0.5 (0.514)	0.12 (0.698)	0.24 (0.524)	0.53 (0.480)	0.89 (0.188)	0.67 (0.583)	1.19 (0.001)	−1.39 (0.05)	2.20 (0.00)	2.14 (0.015)	−0.76 (0.314)	−0.07 (0.852)
Socio-economic and demographic characteristics	0.160	1.870	−0.320	0.750	0.160	−1.500	−2.44	0.830	0.660	0.720	0.040	1.920	0.440	0.030	0.110
Residence	0.000	0.040	0.000	−0.020	0.020	0.040	0.38	0.080	−0.360	0.020	−0.400	−0.120	−0.040	0.000	−0.040
Urban	0 (0.86)	0.02 (0.031)	0 (0.924)	−0.01 (0.391)	0.01 (0.604)	0.02 (0.831)	0.18 (0.943)	0.04 (0.013)	−0.18 (0.126)	0.01 (0.07)	−0.2 (0.026)	−0.06 (0.000)	−0.02 (0.018)	0 (0.799)	−0.02 (0.936)
Rural	0 (0.86)	0.02 (0.031)	0 (0.924)	−0.01 (0.391)	0.01 (0.604)	0.02 (0.831)	0.20 (0.943)	0.04 (0.013)	−0.18 (0.126)	0.01 (0.07)	−0.2 (0.026)	−0.06 (0.000)	−0.02 (0.018)	0 (0.799)	−0.02 (0.936)
Sex of household head	0.080	0.580	0.060	−0.040	0.040	−1.060	−0.78	0.300	2.020	0.340	0.140	0.740	−0.160	−0.020	−0.020
Male	0.04 (0.06)	0.29 (0.000)	0.03 (0.539)	−0.02 (0.538)	0.02 (0.654)	−0.53 (0.833)	−0.33 (0.943)	0.15 (0.168)	1.01 (0.122)	0.17 (0.007)	0.07 (0.065)	0.37 (0.004)	−0.08 (0.115)	−0.01 (0.474)	−0.01 (0.931)
Female	0.04 (0.06)	0.29 (0.000)	0.03 (0.539)	−0.02 (0.538)	0.02 (0.654)	−0.53 (0.833)	−0.39 (0.943)	0.15 (0.168)	1.01 (0.122)	0.17 (0.007)	0.07 (0.065)	0.37 (0.004)	−0.08 (0.115)	−0.01 (0.474)	−0.01 (0.931)
Age group	0.040	0.110	−0.130	−0.120	−0.010	−0.020	0.14	0.490	0.370	−0.010	−0.080	−0.180	0.470	−0.010	0.140
15–19	0.02 (0.059)	0.06 (0.001)	−0.03 (0.176)	−0.07 (0.297)	−0.02 (0.619)	−0.05 (0.825)	0.15 (0.943)	0.42 (0.000)	0.06 (0.316)	−0.01 (0.054)	0.01 (0.138)	0.27 (0.006)	0.45 (0.002)	0 (0.988)	−0.01 (0.934)
20–29	0.01 (0.409)	0.06 (0.026)	0.01 (0.738)	0 (0.985)	0.01 (0.644)	0.01 (0.894)	−0.11 (0.943)	0.01 (0.651)	0.2 (0.119)	0 (0.91)	−0.04 (0.506)	−0.28 (0.006)	0.01 (0.001)	0 (0.566)	0 (0.946)
30–39	0.02 (0.007)	−0.02 (0.000)	−0.08 (0.183)	−0.03 (0.288)	0.01 (0.616)	−0.17 (0.828)	0.12 (0.943)	0.18 (0.000)	0.01 (0.215)	0.05 (0.009)	0.14 (0.089)	0.04 (0.000)	0.13 (0.002)	−0.01 (0.73)	−0.01 (0.934)
40 +	−0.01 (0.65)	0.01 (0.801)	−0.03 (0.208)	−0.02 (0.313)	−0.01 (0.64)	0.19 (0.819)	−0.02 (0.951)	−0.12 (0.002)	0.1 (0.106)	−0.05 (0.515)	−0.19 (0.202)	−0.21 (0.006)	−0.12 (0.008)	0 (0.9)	0.16 (0.935)
Education level	−0.030	0.330	−0.040	0.030	−0.040	0.030	0.12	−0.100	−1.050	−0.210	−0.980	−0.360	−0.030	0.070	−0.090
None	−0.02 (0.14)	0.08 (0.182)	−0.01 (0.763)	−0.01 (0.931)	−0.03 (0.599)	0.01 (0.863)	0.17 (0.943)	−0.06 (0.029)	−0.7 (0.308)	−0.18 (0.148)	−0.58 (0.071)	−0.12 (0.333)	0.01 (0.103)	0.04 (0.577)	0.2 (0.935)
Primary	0 (0.24)	0.02 (0.011)	0 (0.983)	0 (0.548)	−0.01 (0.633)	0 (0.876)	−0.20 (0.943)	0.12 (0.053)	−0.17 (0.382)	0 (0.054)	−0.02 (0.648)	−0.01 (0.352)	−0.01 (0.123)	0 (0.829)	−0.17 (0.934)
Secondary and above	−0.01 (0.633)	0.23 (0.005)	−0.03 (0.674)	0.04 (0.68)	0 (0.683)	0.02 (0.848)	0.15 (0.943)	−0.16 (0.041)	−0.18 (0.548)	−0.03 (0.702)	−0.38 (0.163)	−0.23 (0.111)	−0.03 (0.006)	0.03 (0.651)	−0.12 (0.932)
Standard of living	−0.010	0.030	−0.050	0.030	0.000	0.130	0.28	−0.050	−0.060	−0.090	0.220	−0.070	0.010	−0.030	0.020
1st Quintile	0 (0.816)	0 (0.061)	−0.02 (0.257)	0 (0.365)	0 (0.641)	0.09 (0.828)	0.10 (0.943)	0.02 (0.186)	−0.14 (0.144)	−0.04 (0.003)	0.04 (0.119)	−0.06 (0.253)	0.01 (0.354)	−0.02 (0.571)	0.02 (0.934)
2nd Quintile	0 (0.626)	0.02 (0.071)	0 (0.52)	−0.01 (0.3)	0 (0.731)	−0.01 (0.873)	−0.01 (0.943)	0 (0.67)	0 (0.368)	−0.01 (0.043)	0.01 (0.647)	0 (0.612)	0 (0.412)	0 (0.901)	−0.02 (0.936)
3rd Quintile	0 (0.814)	0.01 (0.009)	0 (0.868)	−0.01 (0.626)	0 (0.725)	0 (0.973)	0.09 (0.943)	0 (0.64)	0.05 (0.121)	0.01 (0.045)	0 (0.677)	0.01 (0.69)	0 (0.687)	0 (0.88)	0.01 (0.934)
4th Quintile	0 (0.082)	0 (0.735)	−0.01 (0.3)	0 (0.406)	0 (0.642)	0.03 (0.834)	0.10 (0.943)	−0.02 (0.472)	0 (0.157)	−0.04 (0.02)	0.01 (0.371)	−0.01 (0.104)	0 (0.356)	−0.01 (0.541)	0 (0.997)
5th Quintile	−0.01 (0.144)	0 (0.41)	−0.02 (0.255)	0.05 (0.336)	0 (0.678)	0.02 (0.846)	0 (0.952)	−0.05 (0.04)	0.03 (0.701)	−0.01 (0.051)	0.16 (0.062)	−0.01 (0.657)	0 (0.345)	0 (0.886)	0.01 (0.936)
Occupation type	0.040	−0.030	-	0.930	0.080	0.160	−1.00	−0.370	−2.200	0.110	0.360	−0.160	−0.070	0.150	0.360
Unemployed	0 (0.706)	−0.22 (0.031)	-	0.45 (0.283)	0.04 (0.661)	−0.11 (0.85)	−0.11 (0.950)	−0.02 (0.919)	-	−0.05 (0.781)	−0.37 (0.019)	−0.45 (0.124)	−0.05 (0.344)	−0.07 (0.619)	0.02 (0.936)
Agricultural	0.03 (0.143)	0.14 (0.209)	-	−0.74 (0.265)	0.02 (0.796)	0.16 (0.822)	−0.97 (0.943)	−0.04 (0.838)	−1.11 (0.213)	−0.01 (0.907)	0.02 (0.012)	0.24 (0.001)	−0.09 (0.077)	0.16 (0.45)	0.15 (0.935)
Domestic	-	0.02 (0.167)	-	-	-	0 (0.872)	-	0.03 (0.263)	0.02 (0.321)	0.01 (0.634)	-	0.02 (0.039)	-	0 (0.534)	-
Trade	0 (0.885)	−0.01 (0.79)	-	1.22 (0.291)	0.01 (0.705)	−0.01 (0.832)	−1.12 (0.940)	−0.06 (0.487)	−0.03 (0.571)	0.12 (0.198)	1.57 (0.006)	0.03 (0.763)	0.09 (0.039)	−0.03 (0.585)	-
Manual labor	0.02 (0.069)	0.05 (0.233)	-	−0.33 (0.265)	0 (0.6)	0.15 (0.81)	0.15 (0.943)	−0.32 (0.196)	−1.26 (0.051)	0 (0.987)	−0.78 (0.015)	0.1 (0.709)	−0.12 (0.192)	0.08 (0.51)	−0.03 (0.925)
Office/service	0.01 (0.276)	0.02 (0.57)	-	0.02 (0.287)	0 (0.669)	−0.05 (0.835)	0.08 (0.943)	0.02 (0.562)	0.28 (0.162)	−0.04 (0.104)	0.34 (0.008)	−0.18 (0.006)	0.01 (0.808)	0.01 (0.703)	0.01 (0.955)
Professional/manager	−0.02 (0.221)	−0.03 (0.665)	-	0.31 (0.258)	0.01 (0.61)	0.02 (0.844)	−0.03 (0.943)	0.02 (0.57)	−0.1 (0.567)	0.08 (0.414)	−0.42 (0.023)	0.08 (0.212)	0.09 (0.022)	0 (0.974)	0.21 (0.935)
Marital status	0.040	0.810	−0.160	−0.060	0.070	−0.780	−1.58	0.480	1.940	0.560	0.780	2.070	0.260	−0.130	−0.260
Never married	0.03 (0.151)	0.4 (0.000)	−0.08 (0.438)	0.03 (0.732)	0.04 (0.617)	−0.26 (0.829)	−0.90 (0.943)	0.38 (0.154)	0.75 (0.219)	0.54 (0.002)	0.65 (0.163)	1.36 (0.000)	0.33 (0.011)	−0.09 (0.471)	−0.21 (0.934)
Married	0 (0.986)	−0.05 (0.013)	−0.12 (0.18)	−0.11 (0.324)	0 (0.762)	−0.03 (0.832)	0.17 (0.943)	−0.6 (0.002)	0.04 (0.423)	−0.16 (0.131)	0.11 (0.679)	−0.07 (0.643)	−0.23 (0.008)	−0.02 (0.585)	0.02 (0.936)
Separated/divorced/widowed	0.01 (0.065)	0.46 (0.000)	0.04 (0.222)	0.02 (0.324)	0.03 (0.621)	−0.49 (0.829)	−0.85 (0.943)	0.7 (0.000)	1.15 (0.135)	0.18 (0.000)	0.02 (0.061)	0.78 (0.000)	0.16 (0.002)	−0.02 (0.461)	−0.07 (0.934)
Sexual behavior factors^f^	0.800	−0.080	−0.060	−0.340	0.010	2.190	2.90	−0.170	−0.010	0.490	−1.240	0.120	1.700	−0.770	−0.140
Sexual behavior risk	0.000	0.000	0.000	0.000	0.000	0.120	0.00	0.000	0.100	0.000	−0.400	0.020	−0.040	−0.020	0.120
No	0 (0.406)	0 (0.255)	0 (0.599)	0 (0.876)	0 (0.612)	0.06 (0.825)	0.00 (0.977)	0 (0.838)	0.05 (0.448)	0 (0.001)	−0.2 (0.192)	0.01 (0.93)	−0.02 (0.163)	−0.01 (0.487)	0.06 (0.933)
Yes	0 (0.406)	0 (0.255)	0 (0.599)	0 (0.876)	0 (0.612)	0.06 (0.825)	0.00 (0.977)	0 (0.838)	0.05 (0.448)	0 (0.001)	−0.2 (0.192)	0.01 (0.93)	−0.02 (0.163)	−0.01 (0.487)	0.06 (0.933)
Premarital sex	−0.040	−0.360	0.040	−0.240	−0.020	1.440	2.85	−1.320	−0.040	−0.240	−1.320	−0.460	0.160	0.040	0.240
No	−0.02 (0.022)	−0.18 (0.007)	0.02 (0.543)	−0.12 (0.29)	−0.01 (0.606)	0.72 (0.821)	1.43 (0.943)	−0.66 (0.000)	−0.02 (0.948)	−0.12 (0.345)	−0.66 (0.042)	−0.23 (0.345)	0.08 (0.01)	0.02 (0.591)	0.12 (0.936)
Yes	−0.02 (0.022)	−0.18 (0.007)	0.02 (0.543)	−0.12 (0.29)	−0.01 (0.606)	0.72 (0.821)	1.42 (0.943)	−0.66 (0.000)	−0.02 (0.948)	−0.12 (0.345)	−0.66 (0.042)	−0.23 (0.345)	0.08 (0.01)	0.02 (0.591)	0.12 (0.936)
Multiple sex partners	0.020	−0.360	−0.020	0.000	−0.020	0.200	−0.06	−0.200	−0.440	0.200	−0.160	−0.280	−0.080	0.100	−0.540
No	0.01 (0.669)	−0.18 (0.003)	−0.01 (0.87)	0 (0.88)	−0.01 (0.607)	0.1 (0.825)	0.03 (0.942)	−0.1 (0.301)	−0.22 (0.437)	0.1 (0.385)	−0.08 (0.743)	−0.14 (0.403)	−0.04 (0.01)	0.05 (0.534)	−0.27 (0.933)
Yes	0.01 (0.669)	−0.18 (0.003)	−0.01 (0.87)	0 (0.88)	−0.01 (0.607)	0.1 (0.825)	0.03 (0.942)	−0.1 (0.301)	−0.22 (0.437)	0.1 (0.385)	−0.08 (0.743)	−0.14 (0.403)	−0.04 (0.01)	0.05 (0.534)	−0.27 (0.933)
Age at first sex	0.820	0.640	−0.080	−0.100	0.050	0.430	−0.01	1.350	0.370	0.530	0.640	0.840	1.660	−0.890	0.040
Never had sex	0.62 (0.000)	0.13 (0.118)	−0.03 (0.336)	0.08 (0.317)	0.03 (0.629)	0.4 (0.829)	−0.05 (0.943)	0.73 (0.000)	0.28 (0.136)	0.17 (0.052)	0.12 (0.426)	0.09 (0.069)	1.24 (0.004)	−0.74 (0.398)	0.02 (0.935)
< 16 years	0.23 (0.000)	0.27 (0.002)	−0.01 (0.633)	−0.02 (0.785)	0.03 (0.678)	0.18 (0.83)	−0.08 (0.943)	0.27 (0.000)	0.5 (0.16)	0.36 (0.001)	0.46 (0.341)	0.07 (0.002)	0.23 (0.015)	−0.56 (0.395)	0.06 (0.934)
16–17	0.19 (0.000)	0.07 (0.089)	−0.03 (0.433)	0.03 (0.48)	0 (0.704)	−0.16 (0.83)	−0.22 (0.944)	0.31 (0.003)	0.09 (0.128)	0.07 (0.097)	0.06 (0.581)	0.41 (0.038)	0.27 (0.009)	−0.14 (0.393)	0.03 (0.936)
18–19	0.06 (0.000)	−0.05 (0.028)	0.02 (0.293)	−0.07 (0.308)	0 (0.628)	−0.08 (0.83)	0.02 (0.943)	0.07 (0.017)	−0.34 (0.142)	0 (0.945)	−0.14 (0.294)	0.03 (0.307)	0.07 (0.015)	0.36 (0.403)	0 (0.944)
20 +	−0.28 (0.000)	0.22 (0.058)	−0.03 (0.589)	−0.12 (0.285)	−0.01 (0.776)	0.09 (0.83)	0.32 (0.943)	−0.03 (0.823)	−0.16 (0.521)	−0.07 (0.665)	0.14 (0.79)	0.24 (0.317)	−0.15 (0.023)	0.19 (0.413)	−0.07 (0.934)
HIV⁄AIDS awareness	0.020	−0.020	0.050	0.050	−0.020	−0.450	0.08	0.270	0.000	−0.070	−0.170	0.170	−0.010	0.000	−0.050
Low	0.01 (0.36)	−0.01 (0.004)	0.02 (0.736)	0.03 (0.462)	−0.01 (0.642)	−0.15 (0.83)	0.04 (0.942)	0.12 (0.029)	−0.02 (0.474)	−0.09 (0.016)	−0.16 (0.245)	0.11 (0.003)	0 (0.809)	0 (0.846)	−0.01 (0.935)
Average	0 (0.178)	0 (0.809)	0 (0.85)	0 (0.917)	0 (0.715)	−0.02 (0.829)	0.00 (0.951)	−0.02 (0.564)	0 (0.241)	0.02 (0.469)	0 (0.263)	0.03 (0.06)	0 (0.937)	0 (0.578)	−0.03 (0.934)
High	0.01 (0.095)	−0.01 (0.002)	0.03 (0.621)	0.02 (0.475)	−0.01 (0.624)	−0.28 (0.828)	0.04 (0.943)	0.17 (0.021)	0.02 (0.423)	0 (0.001)	−0.01 (0.906)	0.03 (0.077)	−0.01 (0.745)	0 (0.591)	−0.01 (0.934)
*The response effect* ^*i*^	0.09 (0.811)	0.34 (0.476)	2.39 (0.000)	2.6 (0.013)	0.76 (0.039)	1.06 (0.028)	3.27 (0.002)	4.16 (0.000)	2.96 (0.051)	1.5 (0.002)	2.18 (0.006)	2.3 (0.006)	9.31 (0.000)	1.44 (0.094)	0.77 (0.106)
Socio-economic and demographic characteristics	0.020	−3.510	−0.360	5.470	0.090	0.390	−0.10	3.000	0.350	−0.930	−7.810	−0.020	3.620	6.220	−0.040
Residence	0.000	−0.100	−0.120	0.110	−0.120	0.060	−0.20	−0.180	0.260	0.010	−0.280	0.030	−0.060	−0.170	−0.020
Urban	0 (0.978)	0.03 (0.498)	−0.28 (0.432)	−0.84 (0.086)	0.04 (0.433)	−0.02 (0.708)	0.63 (0.077)	0.13 (0.599)	−0.22 (0.437)	0.06 (0.702)	0.42 (0.103)	−0.01 (0.909)	0.04 (0.825)	0.34 (0.453)	0.06 (0.646)
Rural	0 (0.978)	−0.13 (0.498)	0.16 (0.432)	0.95 (0.086)	−0.16 (0.433)	0.08 (0.708)	−0.83 (0.077)	−0.31 (0.599)	0.48 (0.437)	−0.05 (0.702)	−0.7 (0.103)	0.04 (0.909)	−0.1 (0.825)	−0.51 (0.453)	−0.08 (0.646)
Sex of household head	0.010	−0.060	−0.070	−0.440	−0.360	−0.120	−0.65	−0.080	−1.140	−0.320	0.450	−0.320	0.070	1.070	0.000
Male	0.01 (0.815)	−0.08 (0.554)	−0.08 (0.909)	−0.51 (0.553)	−0.42 (0.071)	−0.15 (0.599)	−0.75 (0.380)	−0.14 (0.746)	−1.28 (0.183)	−0.38 (0.19)	0.5 (0.254)	−0.38 (0.464)	0.16 (0.697)	1.41 (0.374)	0 (0.984)
Female	0 (0.815)	0.02 (0.554)	0.01 (0.909)	0.07 (0.553)	0.06 (0.071)	0.03 (0.599)	0.09 (0.380)	0.06 (0.746)	0.14 (0.183)	0.06 (0.19)	−0.05 (0.254)	0.06 (0.464)	−0.09 (0.697)	−0.34 (0.374)	0 (0.984)
Age group	0.000	0.010	0.320	0.100	0.010	0.080	0.31	0.800	−0.950	0.000	−0.040	0.180	2.830	0.170	0.090
15–19	0 (0.871)	0.12 (0.417)	−0.14 (0.655)	0.17 (0.703)	0.08 (0.483)	0.2 (0.303)	−0.07 (0.877)	−0.49 (0.377)	0.64 (0.117)	0.44 (0.015)	−0.23 (0.371)	0.67 (0.046)	3.59 (0.000)	−0.63 (0.418)	−0.29 (0.063)
20–29	−0.01 (0.813)	0.25 (0.355)	0.39 (0.159)	0.02 (0.963)	0.05 (0.619)	0.21 (0.249)	0.88 (0.021)	1.72 (0.000)	1.02 (0.092)	0.06 (0.694)	−0.01 (0.952)	0.1 (0.679)	1.32 (0.008)	0.04 (0.91)	0.11 (0.193)
30–39	0 (0.821)	−0.1 (0.372)	0.34 (0.198)	0.13 (0.714)	−0.03 (0.6)	−0.04 (0.61)	0.27 (0.400)	0.32 (0.238)	−1.02 (0.031)	−0.22 (0.018)	0.35 (0.088)	−0.15 (0.348)	−0.97 (0.000)	0.37 (0.469)	0.01 (0.904)
40 +	0.01 (0.814)	−0.26 (0.342)	−0.27 (0.045)	−0.22 (0.272)	−0.09 (0.214)	−0.29 (0.017)	−0.77 (0.009)	−0.75 (0.000)	−1.59 (0.008)	−0.28 (0.016)	−0.15 (0.557)	−0.44 (0.004)	−1.11 (0.000)	0.39 (0.417)	0.26 (0.102)
Education level	0.000	−0.050	−0.380	−0.220	−0.040	0.000	−0.31	1.170	0.040	−0.390	−0.480	0.010	−0.690	0.130	0.180
None	0 (0.814)	0.01 (0.533)	0.01 (0.613)	0.27 (0.454)	−0.1 (0.29)	−0.03 (0.55)	0.03 (0.717)	−0.02 (0.424)	−0.25 (0.346)	0.08 (0.229)	−0.87 (0.034)	−0.04 (0.449)	0.08 (0.348)	−0.12 (0.574)	−0.05 (0.187)
Primary	0 (0.816)	0.04 (0.705)	−0.05 (0.759)	0.28 (0.293)	0.02 (0.823)	−0.03 (0.884)	0.24 (0.620)	0.29 (0.456)	−0.16 (0.855)	0 (0.974)	0.16 (0.227)	−0.26 (0.419)	0.26 (0.365)	0.19 (0.58)	0.2 (0.145)
Secondary and above	0 (0.899)	−0.1 (0.453)	−0.34 (0.581)	−0.77 (0.119)	0.04 (0.344)	0.06 (0.519)	−0.58 (0.318)	0.9 (0.46)	0.45 (0.278)	−0.47 (0.084)	0.23 (0.262)	0.31 (0.203)	−1.03 (0.041)	0.06 (0.886)	0.03 (0.849)
Standard of living	0.000	0.020	0.460	0.510	0.010	−0.080	0.130	0.060	−0.310	0.000	−0.140	0.020	−0.150	0.130	−0.360
1st Quintile	0 (0.985)	0.01 (0.828)	−0.15 (0.115)	−0.52 (0.082)	−0.08 (0.483)	−0.15 (0.178)	0.36 (0.281)	−0.12 (0.585)	−0.27 (0.608)	−0.03 (0.789)	0.16 (0.37)	0.17 (0.252)	−0.41 (0.091)	−0.16 (0.673)	−0.62 (0.086)
2nd Quintile	0 (0.822)	−0.07 (0.4)	−0.09 (0.383)	−0.34 (0.141)	0.09 (0.443)	−0.1 (0.247)	−0.06 (0.742)	0.04 (0.848)	−0.69 (0.098)	−0.08 (0.331)	0.01 (0.941)	−0.02 (0.85)	−0.12 (0.511)	−0.04 (0.864)	−0.62 (0.065)
3rd Quintile	0 (0.818)	0.03 (0.539)	0 (0.974)	0.16 (0.594)	0 (0.952)	−0.03 (0.743)	−0.17 (0.396)	0.27 (0.261)	0.47 (0.135)	0.13 (0.094)	0.13 (0.388)	−0.06 (0.665)	−0.01 (0.98)	−0.02 (0.899)	−0.68 (0.057)
4th Quintile	0.000 (0.828)	0.02 (0.634)	0.27 (0.218)	0.000 (0.999)	−0.01 (0.766)	0.11 (0.165)	−0.01 (0.946)	0.14 (0.571)	0.18 (0.411)	0.07 (0.524)	−0.02 (0.839)	−0.09 (0.427)	−0.28 (0.223)	0.44 (0.425)	0.72 (0.05)
5th Quintile	0.000 (0.842)	0.03 (0.616)	0.43 (0.133)	1.21 (0.026)	0.01 (0.762)	0.09 (0.289)	0.01 (0.980)	−0.27 (0.277)	0 (0.998)	−0.09 (0.39)	−0.42 (0.107)	0.02 (0.926)	0.67 (0.011)	−0.09 (0.635)	0.84 (0.055)
Occupation type	0.010	−3.280		4.970	0.450	0.260	0.010	0.270	2.470	0.010	−0.430	−0.020	0.000	5.260	0.150
Unemployed	0 (0.849)	−0.61 (0.349)	-	1.79 (0.035)	0.06 (0.183)	−0.04 (0.518)	0.04 (0.901)	−0.35 (0.368)	-	−0.08 (0.571)	0.27 (0.319)	−0.07 (0.633)	−0.04 (0.937)	1.23 (0.311)	−0.04 (0.71)
Agricultural	0.01 (0.814)	−0.72 (0.346)	-	2.11 (0.037)	0.44 (0.131)	0.31 (0.339)	−0.23 (0.666)	0.02 (0.944)	1.59 (0.148)	−0.02 (0.898)	−0.29 (0.403)	−0.21 (0.554)	0.07 (0.628)	3.16 (0.365)	0.19 (0.202)
Domestic	-	0.09 (0.346)	-	-	-	0.02 (0.586)	-	−0.06 (0.176)	−0.06 (0.368)	−0.05 (0.143)	-	0 (0.823)	-	−0.05 (0.342)	-
Trade	0 (0.818)	−0.26 (0.348)	-	0.47 (0.052)	−0.03 (0.313)	0.08 (0.077)	0.24 (0.129)	0.14 (0.227)	0.61 (0.061)	0.05 (0.427)	−0.19 (0.017)	0.23 (0.042)	0.07 (0.403)	0.24 (0.439)	-
Manual labor	0 (0.813)	−1.36 (0.346)	-	0 (0.999)	0.03 (0.368)	−0.07 (0.57)	0.04 (0.869)	0.38 (0.309)	0.53 (0.315)	0.03 (0.861)	−0.15 (0.204)	0.06 (0.76)	0.04 (0.872)	0.14 (0.527)	0.06 (0.47)
Office/service	0 (0.815)	−0.21 (0.352)	-	1 (0.025)	−0.01 (0.253)	−0.04 (0.092)	−0.12 (0.135)	−0.08 (0.228)	−0.19 (0.294)	0.12 (0.029)	0.05 (0.003)	0.02 (0.736)	0.01 (0.966)	0.34 (0.38)	−0.06 (0.122)
Professional/manager	0 (0.819)	−0.21 (0.346)	-	−0.4 (0.015)	−0.04 (0.126)	0 (0.851)	0.04 (0.740)	0.22 (0.059)	−0.01 (0.953)	−0.04 (0.435)	−0.12 (0.068)	−0.05 (0.32)	−0.15 (0.308)	0.2 (0.43)	0 (0.946)
Marital status	0.000	−0.050	−0.570	0.440	0.140	0.190	0.630	0.960	−0.020	−0.240	−6.890	0.080	1.620	−0.370	−0.080
Never married	0 (0.825)	0.13 (0.465)	0.25 (0.473)	0.5 (0.532)	0.02 (0.868)	0.56 (0.095)	2.18 (0.002)	3.79 (0.000)	0.25 (0.569)	0.04 (0.851)	−2.7 (0.004)	0.73 (0.175)	2.6 (0.001)	1.17 (0.389)	−0.01 (0.954)
Married	0 (0.821)	−0.17 (0.424)	−0.92 (0.015)	0 (0.995)	0.13 (0.401)	−0.36 (0.162)	−1.39 (0.036)	−2.68 (0.000)	−0.2 (0.885)	−0.3 (0.168)	−4.76 (0.001)	−0.62 (0.216)	−0.96 (0.001)	−1.51 (0.395)	−0.08 (0.614)
Separated/divorced/widowed	0 (0.885)	−0.01 (0.717)	0.1 (0.091)	−0.06 (0.456)	−0.01 (0.272)	−0.01 (0.272)	−0.17 (0.009)	−0.15 (0.004)	−0.07 (0.46)	0.02 (0.318)	0.57 (0.001)	−0.03 (0.33)	−0.02 (0.631)	−0.03 (0.679)	0.01 (0.674)
Sexual behavioral factors^f^	0.030	0.200	0.200	−0.640	0.440	−0.490	1.47	1.320	0.960	−0.650	0.330	−0.120	0.490	−2.550	−0.190
Sexual behavior risk	0.020	0.180	−0.020	−0.850	0.700	−0.120	−0.320	1.880	−0.240	−0.520	−0.120	−0.990	1.160	0.000	−0.250
No	0.02 (0.813)	0.21 (0.436)	0.08 (0.65)	−1.12 (0.183)	0.72 (0.066)	−0.12 (0.858)	−0.36 (0.703)	1.98 (0.081)	−0.3 (0.743)	−0.64 (0.019)	−0.15 (0.772)	−1.13 (0.1)	1.4 (0.015)	0 (0.99)	−0.36 (0.18)
Yes	0 (0.813)	−0.03 (0.436)	−0.1 (0.65)	0.27 (0.183)	−0.02 (0.066)	0 (0.858)	0.04 (0.703)	−0.1 (0.081)	0.06 (0.743)	0.12 (0.019)	0.03 (0.772)	0.14 (0.1)	−0.24 (0.015)	0 (0.99)	0.11 (0.18)
Premarital sex	0.000	0.070	0.290	−0.130	0.010	0.080	−0.010	0.000	−0.590	−0.210	0.090	−0.010	−0.010	−0.170	−0.080
No	0.000 (0.822)	−0.08 (0.419)	−0.06 (0.424)	0.06 (0.801)	0.01 (0.899)	0.27 (0.086)	0.01 (0.976)	−0.03 (0.927)	0.58 (0.112)	0.2 (0.08)	−0.07 (0.615)	0.000 (0.979)	0.01 (0.971)	0.06 (0.712)	0.05 (0.412)
Yes	0.000 (0.822)	0.15 (0.419)	0.35 (0.424)	−0.19 (0.801)	0 (0.899)	−0.19 (0.086)	−0.02 (0.976)	0.03 (0.927)	−1.17 (0.112)	−0.41 (0.08)	0.16 (0.615)	−0.01 (0.979)	−0.02 (0.971)	−0.23 (0.712)	−0.13 (0.412)
Multiple sex partners	−0.010	−0.080	−0.070	0.020	−0.440	0.080	0.600	0.170	−0.040	0.050	0.140	0.510	0.130	0.160	0.200
No	−0.01 (0.81)	−0.12 (0.53)	0.46 (0.022)	0.62 (0.136)	−0.46 (0.116)	0.09 (0.756)	0.96 (0.104)	0.24 (0.69)	−0.07 (0.935)	0.17 (0.308)	0.3 (0.431)	0.68 (0.15)	0.27 (0.551)	0.73 (0.422)	0.35 (0.133)
Yes	0 (0.81)	0.04 (0.53)	−0.53 (0.022)	−0.6 (0.136)	0.02 (0.116)	−0.01 (0.756)	−0.36 (0.104)	−0.07 (0.69)	0.03 (0.935)	−0.12 (0.308)	−0.16 (0.431)	−0.17 (0.15)	−0.14 (0.551)	−0.57 (0.422)	−0.15 (0.133)
Age at first sex	0.020	0.030	0.000	0.320	0.170	−0.530	1.200	−0.730	1.830	0.030	0.220	0.370	−0.790	−2.540	−0.060
Never had sex	0.09 (0.814)	−0.14 (0.412)	−0.03 (0.854)	−0.57 (0.327)	0.16 (0.307)	−0.87 (0.068)	−1.17 (0.026)	−2.91 (0.001)	−0.31 (0.371)	−0.21 (0.45)	−0.41 (0.161)	−0.58 (0.169)	−1.49 (0.203)	2.39 (0.347)	0.09 (0.398)
< 16 years	−0.01 (0.814)	0.07 (0.387)	0.31 (0.399)	−0.15 (0.704)	−0.02 (0.251)	0.15 (0.101)	0.76 (0.117)	0.28 (0.029)	0.89 (0.262)	0.1 (0.342)	0.1 (0.507)	0.48 (0.126)	0.27 (0.105)	−0.88 (0.347)	−0.03 (0.785)
16–17	−0.01 (0.815)	0.05 (0.479)	−0.31 (0.138)	0.17 (0.592)	−0.04 (0.142)	0.07 (0.227)	0.58 (0.073)	0.35 (0.053)	0.64 (0.358)	0.19 (0.148)	0.16 (0.343)	0.14 (0.319)	−0.19 (0.324)	−1.53 (0.344)	−0.1 (0.222)
18–19	−0.02 (0.814)	0.09 (0.404)	−0.14 (0.333)	0.67 (0.119)	0.03 (0.565)	0.14 (0.111)	0.35 (0.323)	0.53 (0.03)	0.34 (0.58)	−0.02 (0.89)	0.3 (0.146)	0.12 (0.464)	0.55 (0.043)	−1.87 (0.351)	0.01 (0.81)
20 +	−0.03 (0.815)	−0.04 (0.586)	0.17 (0.115)	0.2 (0.512)	0.04 (0.743)	−0.02 (0.863)	0.68 (0.060)	1.02 (0.014)	0.27 (0.663)	−0.03 (0.778)	0.07 (0.807)	0.21 (0.361)	0.07 (0.814)	−0.65 (0.389)	−0.03 (0.646)
HIV⁄AIDS awareness	0.010	−0.030	0.070	0.570	0.010	−0.020	0.130	0.120	0.130	−0.010	0.040	0.030	−0.040	−0.030	0.090
Low	0.000 (0.815)	0.000 (0.961)	−0.07 (0.556)	−0.45 (0.048)	−0.05 (0.408)	−0.07 (0.551)	0.24 (0.429)	−0.19 (0.445)	0.92 (0.07)	−0.34 (0.014)	−0.15 (0.346)	0.01 (0.94)	0.01 (0.966)	0.26 (0.477)	0.96 (0.118)
Average	0 (0.819)	−0.07 (0.449)	0.02 (0.923)	1.34 (0.009)	0.09 (0.229)	−0.01 (0.893)	0.06 (0.816)	0 (0.997)	−0.76 (0.064)	−0.11 (0.347)	0.43 (0.085)	0.15 (0.375)	−0.1 (0.732)	−0.52 (0.373)	0.67 (0.049)
High	0.01 (0.814)	0.04 (0.466)	0.12 (0.58)	−0.32 (0.388)	−0.03 (0.716)	0.06 (0.437)	−0.18 (0.314)	0.31 (0.329)	−0.03 (0.931)	0.44 (0.004)	−0.24 (0.242)	−0.13 (0.351)	0.05 (0.794)	0.23 (0.509)	−1.54 (0.061)
Constant	0.04 (0.812)	3.68 (0.357)	2.48 (0.018)	−2.78 (0.309)	0.21 (0.541)	1.19 (0.183)	1.77 (0.242)	−0.3 (0.885)	1.52 (0.585)	3.09 (0.000)	9.61 (0.001)	2.4 (0.034)	5.22 (0.000)	−2.19 (0.477)	0.92 (0.126)

*P*-values are reported in parenthesis; They are testing variables’ contribution to gender inequality in HIV/AIDS in each country

^a^Domestic category not collected

^b^Occupation type variable not collected

^c^Unemployed category not collected

^d^Trade and domestic category not collected

^e^The overall absolute contribution of a given variable is equal to the sum of absolute contribution of its categories; the absolute contribution of socio-demographic characteristics, sexual behaviours, and awareness of HIV/AIDS is determined by summing the absolute contributions of all variables included in each category. The absolute contribution of a given variable to a given component was calculated as follow: the absolute difference of HIV/AIDS prevalence between women and men explained by this component multiplied by the relative contribution (in percentage) of this variable to this absolute difference in HIV/AIDS prevalence

^f^Sexual behavioural factors are not highly correlated

^g^Several values of dichotomous variables for the “difference due to distribution” component became identical after rounding the estimated values

^h^Represent the contribution to gender inequalities in HIV/AIDS prevalence due to gender differences in the distributions of observable HIV/AIDS risk factors between women and men

^i^Reflect the contribution to gender inequalities in HIV/AIDS due to gender differences in the effects of measured HIV/AIDS risk factors, as well as unmeasured factors not included in the model

In Ghana (2003) and Uganda (2011), it was the composition effect that explained gender inequalities in HIV/AIDS prevalence. Differences in the distribution of sexual behaviors explained 74.1 % of the excess in HIV seropositivity among women in Ghana. For example, in Ghana 24 % of men reported that they had never had sex compared to 15.5 % of women; results from the BO decomposition imply a 57.4 % decrease in the gender inequality in HIV/AIDS if Ghanaian women and men were equally likely to report not having sex. In Uganda, the differential distribution of socio-demographic characteristics between women and men was responsible for 89 % of the gender inequality in HIV/AIDS. The differential distributions of marital status, particularly being separated, divorced, or widowed, explained 38.6 % and 21.9 % of excess HIV/AIDS seropositivity among women in Ghana and Uganda, respectively. In both countries, the differential distributions of premarital sex between men (54.3 % in Ghana, 66 % in Uganda) and women (43.1 % in Ghana, 45.5 % in Uganda) reduced gender gaps in HIV/AIDS prevalence.

In countries where both response and composition effects explained gender inequalities in HIV/AIDS prevalence (Cameroon, Guinea, Malawi, and Swaziland), age contributed to gender inequalities in HIV/AIDS prevalence. Results from the BO decomposition imply that setting the distributions of age to be the same for women and men and equalizing the effect of age on HIV/AIDS prevalence would lead to a 28.8 % decrease in the excess prevalence of HIV/AIDS among women in Swaziland. However, doing so in Guinea and Cameroon would increase these inequalities by 15.2 %. The differential effect of age did not contribute to gender inequality in HIV/AIDS in Malawi. In all four of these countries, the constant term, representing the effects of unmeasured factors, made the largest contribution to gender inequalities.

Seven countries were surveyed twice between 2003 and 2012. In six of the seven earlier surveys there was a significant difference in the prevalence of HIV/AIDS between women and men. We examined the sources of gender inequality in HIV/AIDS in these six surveys, fielded between 2004 and 2006 (Additional file 1: Table S2). Similar to our main findings, analyses of these six additional surveys showed that, in most countries, inequalities in HIV/AIDS prevalence between men and women were attributable to the differences in the responses to HIV/AIDS risk factors (Additional file 1: Table S3). The response effect of unmeasured characteristics made the largest contribution to gender inequalities in HIV/AIDS prevalence in most of these six countries. Additionally, the main sources of gender inequalities in HIV/AIDS within countries changed over time in Cameroon, Ethiopia, Malawi and Zimbabwe.

## Discussion

The global burden of HIV/AIDS varies considerably between countries, with those in southern Africa being most affected by the pandemic [60]. We estimated the absolute difference in HIV/AIDS prevalence comparing women to men in 21 SSA countries and identified sources of gender inequalities. Descriptive analyses showed that gender inequalities in HIV/AIDS prevalence were most pronounced in the south-east region of SSA, where socioeconomic inequalities in HIV/AIDS are also greater relative to other SSA countries [61]. In these countries in particular, gender inequalities in HIV/AIDS prevalence were primarily explained by the differential effects of HIV/AIDS risk factors for men and women rather than by the differential distributions of these characteristics. Reducing gender inequalities might be essential to efforts to eliminate HIV/AIDS [62].

There are gender inequalities in the prevalence of HIV/AIDS in SSA across countries. Our analyses showed that women had a higher prevalence of HIV/AIDS than men in at least three-quarters of the countries surveyed. Furthermore, a comparison of gender inequalities within countries surveyed on two occasions since 2003 suggests that these inequalities are persistent over time. Consistent with earlier work [63], our results showed that gender inequalities in HIV/AIDS prevalence were larger in magnitude in countries with a greater burden of HIV/AIDS, particularly countries situated in south-east SSA, including Malawi, Mozambique, Swaziland, Zambia, and Zimbabwe. A higher probability of HIV transmission [64], as well as greater community viral load (CVL) [65], may contribute to greater prevalence of HIV/AIDS in this region. A systematic review of observational studies by Boily and colleagues [64] suggested that regional differences might be explained by variations in contraceptive practices, differential burden of viral subtypes, and interaction with other infectious diseases, among other factors. Additionally, a recent study by Abu-Raddad and colleagues [65] indicated that viral load is higher in SSA than other regions, and it is highest in southern and east Africa. Community viral load may be a central driver of the HIV epidemic in SSA, where it may reflect, among other factors, the high burden of co-infections such as malaria, tuberculosis and other tropical diseases or the preponderance of HIV-1 subtype C infection. Other work suggests that the level of wealth inequality in SSA region is associated with HIV prevalence [65–67].

Results from our decomposition analysis showed that the sources of gender inequality in HIV/AIDS vary across countries. In most of the countries surveyed, gender inequalities in HIV/AIDS were primarily explained by differences in the effects of risk factors—both measured (i.e. socio-demographic characteristics, sexual behaviours, and HIV⁄AIDS awareness) and unmeasured in our model—on HIV/AIDS seropositivity for women and men. Gender-related constraints, including women’s limited control of resources, may decrease women’s ability to protect themselves against diseases [68] and explain our observations. For example, with respect to measured socio-demographic characteristics, the differential effects of occupation contributed to the disproportionate burden of HIV/AIDS among women in SSA. The same occupational classes may be associated with different risks for HIV/AIDS for women compared to men. For example, research indicates that unemployed women generally face poorer job prospects than unemployed men [69]. Further, unemployed women are more economically dependent on their male partners and have fewer alternatives to protect themselves against disease transmission [68]. Similarly, marital status has different implications for women and men in many contexts because of cultural restrictions on women’s autonomy in the public sphere. These constraints may contribute to gender inequalities in HIV/AIDS by reducing the capacity of unmarried women to engage in equitable relationships and negotiate safe sexual practices (e.g. condom use) with their partners [70, 71], which increases the probability of HIV infection. Other research showed that women who exited an abusive marriage were likely to enter another one with new risks [72], or enter domestic service, which is associated with workplace violence [73].

In a subset of countries, differences in the distributions of sexual behaviors, including age at first sex and premarital sex, between women and men were the main factors contributing to gender inequalities in HIV/AIDS. The age of first sex was lower, on average, for women compared to men, suggesting they were at higher risk of HIV infection due to a longer risk period [74]. Consistent with earlier work [75–78], interventions that delay the age at which women experience intercourse might reduce gender inequalities in HIV/AIDS. Indeed, it has been shown that a longer duration of premarital sex relative to the duration of marriage was associated with an increased odds of HIV infection and other sexually transmitted diseases [79]. In our sample, women were less likely to have premarital sex compared to men (see descriptive analysis in Additional file 1: Table S1). This likely protected women against HIV/AIDS. Moreover, our analysis showed that in countries where composition and response sources of gender inequalities in HIV/AIDS played a significant role (e.g. Cameroon, Guinea, Malawi and Swaziland), the differential effects of unmeasured factors made a large and significant contribution. Unmeasured factors that might contribute to gender inequalities in HIV/AIDS include power processes through couple communication and collaboration [80], lack of female empowerment and limited access to health resources [40], social support [81], migration [82], and lack of an enabling environment for women [83]. Also, several unmeasured biological mechanisms might increase women’s risk of contracting HIV [84]. First, research indicates that during sexual intercourse women have a greater mucosal surface area exposed to infectious fluid for longer periods and are more likely to face increased tissue injury [85]. Second, sexually transmitted infectious diseases increase the risk of contracting HIV, particularly for women, because these infections are often asymptomatic and untreated [85–87]. Third, women have a window of vulnerability after ovulation in which the potential for viral infectivity in the female reproductive tract is increased [88]. Further work is required to understand how the differential effects of risk factors contribute to gender inequalities in HIV/AIDS.

Our study is subject to some potential limitations. *First*, although the DHS/AIS provide nationally representative estimates of HIV prevalence, our results could be biased due to the voluntary nature of the HIV test. Nevertheless, prior studies [24, 89] showed that non-response was unlikely to bias national estimates of prevalence from the DHS. Further, it has been shown that non-response is more likely to be random than selective in the DHSs [3]. *Second*, the cross-sectional nature of data cannot establish temporality between risk factors and outcome status, making it impossible to rule out reverse causality—our findings should therefore be interpreted as associations rather than causal estimates of the impact of intervening on HIV/AIDS risk factors. Third, because we used individual-level data we could not quantify the contribution of structural factors (e.g., CVL, wealth inequality) to gender inequalities in HIV/AIDS. In brief, this is because structural factors operate at the aggregate-level and cannot be used to decompose individual-level gender inequalities in HIV/AIDS prevalence. We also could not incorporate biological factors in our analyses, as this information was not available in our datasets. Finally, HIV risk factors were self-reported and may be reported with error; for example, individuals might misreport sexual behaviors [90].

Caveats considered, the results of this study have some useful implications for future research and for potential interventions targeting gender inequalities in HIV/AIDS in SSA. First, in the majority of countries it was the differential effects of measured and unmeasured HIV/AIDS risk factors that contributed to gender inequalities. Further research, including sub-national research and mixed methods approaches, is needed to identify these risk factors and elucidate how they interact with gender to exacerbate differences in the burden of HIV/AIDS between women and men. Second, when these processes are better understood, our findings suggest that country-specific interventions and preventive programs based on the sources of gender inequalities in each context [37, 38] will be needed to mitigate gender inequalities in HIV/AIDS in SSA. Given the different patterns that we observed in the sources and factors contributing to gender inequalities across countries, general interventions to reduce gender inequalities in HIV/AIDS may not be effective [37]. Prior work [91–97] has proposed a structural approach to HIV prevention which takes into account the main dimensions of women’s empowerment; examining whether interventions designed to empower women reduce inequalities in HIV/AIDS is a fruitful area for further research.

## Conclusions

Using the most recent available data we measured gender inequalities in HIV/AIDS prevalence in the SSA region and identified sources and factors contributing to these inequalities. We found three unique patterns: i) countries where gender inequality in HIV/AIDS prevalence was due to differences in the distribution of risk factors between men and women, ii) countries where gender inequalities in HIV/AIDS prevalence were due to differences in the effects of risk factors on prevalence of HIV/AIDS for women and men, and iii) countries where the combination of risk factors being distributed differently and having differential effects for men and women explained gender inequalities in HIV/AIDS prevalence. In countries (e.g. Cameroon, Guinea, Malawi, and Swaziland) where unmeasured characteristics substantially contributed to gender inequalities, future work is required to understand the factors underpinning inequalities. Moreover, by adding to extant knowledge concerning the determinants of gender inequalities in HIV/AIDS in SSA, our study can help prioritize interventions to tackle gender inequalities in HIV/AIDS prevalence.

## Additional file

Additional file 1: This document provides three tables titled “**Table S1.** Sample characteristics by gender and surveys”, “**Table S2.** Results from Blinder-Oaxaca decomposition analysis of gender inequalities in HIV/AIDS prevalence using earlier DHSs conducted between 2004 and 2006, for countries surveyed twice between 2003 and 2012” and “**Table S3.** Results from Blinder-Oaxaca decomposition analysis of gender inequalities in HIV/AIDS prevalence using earlier DHSs conducted between 2004 and 2006, for countries surveyed twice between 2003 and 2012”. (DOCX 110 kb)
