# Supplementary material for: What explains gender inequalities in HIV/AIDS prevalence in sub-Saharan Africa? Evidence from the demographic and health surveys
Source: BMC Public Health. 2016 Nov 3;16:1136. doi: 10.1186/s12889-016-3783-5 (PMC5095963; doi:10.1186/s12889-016-3783-5)
Supplement: Additional file 1: — This document provides three tables titled “Table S1. Sample characteristics by gender and surveys”, “Table S2. Results from Blinder-Oaxaca decomposition analysis of gender inequalities in HIV/AIDS prevalence using earlier DHSs conducted between 2004 and 2006, for countries surveyed twice between 2003 and 2012” and “Table S3. Results from Blinder-Oaxaca decomposition analysis of gender inequalities in HIV/AIDS prevalence using earlier DHSs conducted between 2004 and 2006, for countries surveyed twice between 2003 and 2012”. (DOCX 110 kb) [file 12889_2016_3783_MOESM1_ESM.docx]

**Additional Files: Appendices**

**Table A1**: Sample characteristics by gender and surveys

|  |  | **Burkina Faso**  **2003** | | **Burkina Faso**  **2010** | | **Cameroon**  **2004** | | **Congo Brazzaville 2009†** | | **Côte d’Ivoire**  **2005** | | **Ghana**  **2003** | |
| --- | --- | --- | --- | --- | --- | --- | --- | --- | --- | --- | --- | --- | --- |
|  |  | **Male** | **Female** | **Male** | **Female** | **Male** | **Female** | **Male** | **Female** | **Male** | **Female** | **Male** | **Female** |
|  | **Sample Size** | 3341 | 4189 | 3095 | 3811 | 2234 | 3020 | 3075 | 3849 | 4774 | 5969 | 6333 | 8711 |
| ***Socio-economic and Demographic Characteristics*** | | | | | | | | | | | | | |
|  | **Residence** |  |  |  |  |  |  |  |  |  |  |  |  |
|  | Rural | 75.9 | 77.2 | 71.1 | 73.2** | 42.8 | 45.0 | 36.4 | 37.4 | 52.91 | 52.0 | 55.1*** | 51.6 |
|  | Urban | 24.1 | 22.8 | 28.9 | 26.8 | 57.2* | 56.0 | 63.6 | 62.6 | 47.09 | 48.0 | 44.9 | 48.4 |
|  | **Sex of household head** | |  |  |  |  |  |  |  |  |  |  |  |
|  | Male | 96.4 | 90.5 | 96.0 | 89.1 | 88.4 | 74.8 | 90.9 | 71.9 | 88.6 | 75.5 | 86.9 | 62.8 |
|  | Female | 3.6 | 9.5*** | 4.0 | 10.9*** | 11.6 | 25.2*** | 9. 1 | 28.1*** | 11.4 | 24.6 | 13.1 | 37.2*** |
|  | **Age group (in years)** | |  |  |  |  |  |  |  |  |  |  |  |
|  | 15-19 | 25.1 | 23.4 | 19.8 | 19.3 | 23.7 | 24.9 | 19.3 | 19.8 | 20.5 | 23.2 | 23.2** | 20.3 |
|  | 20-29 | 27.2 | 33.4*** | 27.4 | 37.0*** | 33.2 | 37.8*** | 32.5 | 38.8 | 40.0 | 40.2 | 28.6 | 34.7*** |
|  | 30-39 | 21.7 | 25.0* | 23.8 | 27.4*** | 20.4 | 23.0** | 30.7 | 25.4 | 24.0 | 22.7 | 22.0 | 26.5 |
|  | 40 + | 26.0 | 18.2*** | 28.0*** | 16.3 | 22.7*** | 14.3 | 17.4 | 16.0 | 15.5 | 13.9 | 26.3*** | 18.5 |
|  | **Education level** | |  |  |  |  |  |  |  |  |  |  |  |
|  | None | 64.6 | 79.4*** | 61.4 | 74.0*** | 11.3 | 22.0*** | 1.9 | 5.7*** | 32.4 | 54.1*** | 17.3 | 28.2*** |
|  | Primary | 18.9*** | 12.3 | 19.8*** | 13.8 | 37.1 | 39.1 | 18.7 | 27.8*** | 26.3 | 27.5 | 15.9 | 20.2*** |
|  | Secondary and above | 16.5*** | 8.3 | 18.8*** | 12.2 | 51.6*** | 38.9 | 79.4*** | 66.5 | 41.3*** | 18.4 | 66.8*** | 51.6 |
|  | **Standard of living** | |  |  |  |  |  |  |  |  |  |  |  |
|  | 1^st^ Quintile (lowest) | 21.9 | 21.1 | 21.9 | 22.3 | 26.0 | 27.1 | 9.3 | 11.6*** | 16.0 | 15.8 | 19.4** | 17.5 |
|  | 2^nd^ Quintile | 21.2 | 21.8 | 21.1 | 20.6 | 12.8 | 14.2 | 13.7 | 13.1 | 15.0 | 15.6 | 17.1 | 16.1 |
|  | 3^rd^ Quintile | 18.0 | 19.0 | 18.3 | 19.1 | 18.5 | 17.8 | 16.0 | 17.5 | 19.8* | 16.4 | 20.1 | 20.7 |
|  | 4^th^ Quintile | 17.6 | 18.3 | 18.5 | 18.5 | 20.7* | 19.0 | 28.6 | 27.4 | 23.6 | 22.6 | 24.6 | 23.9 |
|  | 5^th^ Quintile | 21.3 | 19.7 | 20.2 | 19.5 | 22.0 | 21.9 | 32.4* | 30.4 | 25.6 | 29.5* | 18.8 | 21.8*** |
|  | **Occupation type** | |  |  |  |  |  |  |  |  |  |  |  |
|  | Agricultural | 61.0 | 64.4 | 66.8*** | 47.3 | 33.1 | 32.3 | -- | -- | 37.9*** | 20.0 | 40.1*** | 28.3 |
|  | Unemployed | 16.9** | 12.4 | 4.8 | 21.9*** | 25.7 | 38.1*** | -- | -- | 23.5 | 32.5*** | 22.7 | 22.3 |
|  | Domestic | 3.3 | 2.6 | 0.1 | 1.3*** | 0.4* | 0.1 | -- | -- |  |  |  |  |
|  | Trade | 6.4 | 16.2*** | 11.0 | 20.8*** | 0.5 | 0.3 | -- | -- | 7.8 | 31.0*** | 5.5 | 26.3*** |
|  | Manual labor | 2.6 | 1.8 | 11.4*** | 6.8 | 24.3 | 24.9 | -- | -- | 15.4*** | 2.0 | 19.6*** | 13.3 |
|  | Office / service | 6.6*** | 1.6 | 1.9*** | 0.8 | 11.7*** | 3.4 | -- | -- | 14.0 | 14.4 | 4.2 | 7.5*** |
|  | Professional / manager | 3.2*** | 1.0 | 4.0*** | 1.1 | 4.3*** | 0.9 | -- | -- | 1.5*** | 0.1 | 7.9*** | 2.2 |
|  | **Marital status** | |  |  |  |  |  |  |  |  |  |  |  |
|  | Married | 55.5 | 76.0*** | 63.7 | 79.2*** | 50.4 | 68.4*** | 51.3 | 59.2*** | 42.7 | 58. 1*** | 53.0 | 62.6*** |
|  | Never married | 42.6*** | 20.0 | 34.3*** | 17.6 | 40.4*** | 23.2 | 40.1*** | 27.3 | 51.2*** | 32.9 | 41.6*** | 28.3 |
|  | Separated / divorced / widowed | 1.9 | 4.0*** | 2.0 | 3.2*** | 9.2 | 8.5 | 8.6 | 13.5*** | 6.1 | 9.0* | 5.4 | 9.1*** |
| ***Sexual Behaviors*** | | | | | | | | | | | | | |
|  | **sexual behavior risk** | |  |  |  |  |  |  |  |  |  |  |  |
|  | No | 93.6 | 96.8 | 95.8 | 96.5 | 83.9 | 88.6 | 46.1 | 48.5 | 80.3 | 82.6 | 90.2 | 89.9 |
|  | Yes | 6.4*** | 3.2 | 4.2 | 3.5 | 16.1*** | 11.4 | 53.9 | 51.5 | 19.7 | 17.4 | 9.8 | 10.1 |
|  | **Premarital sex** | |  |  |  |  |  |  |  |  |  |  |  |
|  | No | 51.0 | 77.4 | 47.5 | 83.4 | 36.0 | 58.7 | 15.4 | 26.4 | 22.2 | 41.7 | 45.7 | 56.9 |
|  | Yes | 49.0*** | 22.6 | 52.5*** | 16.6 | 64.0*** | 41.3 | 84.6*** | 73.6 | 77.8*** | 58.3 | 54.3*** | 43.1 |
|  | **Multiple sex partners** | |  |  |  |  |  |  |  |  |  |  |  |
|  | No | 77.7 | 93.0 | 82.0 | 94.0 | 54.7 | 78.2 | 46.4 | 70.0 | 50.7 | 57.3 | 77.5 | 86.1 |
|  | Yes | 22.3*** | 7.0 | 18.0*** | 6.0 | 45.3 | 21.8 | 53.59*** | 30.0 | 49.3** | 42.7 | 22.5*** | 13.9 |
|  | **Age at first sex** |  |  |  |  |  |  |  |  |  |  |  |  |
|  | 20 + | 39.6*** | 7.2 | 46.1*** | 13.4 | 22.5*** | 5.8 | 8.8** | 5.0 | 18.6*** | 6.6 | 34.4*** | 17.4 |
|  | Never had sex | 23.6*** | 13.9 | 20.2*** | 12.2 | 16.3*** | 12.2 | 8.0* | 6.0 | 12.3* | 8.8 | 24.0*** | 15.5 |
|  | < 16 years | 9.6 | 22.8*** | 4.7 | 22.6*** | 20.0 | 41.1*** | 43.9 | 46.9 | 27.8 | 42.3*** | 10.1 | 21.1*** |
|  | 16-17 | 12.5 | 37.7*** | 11.0 | 31.9*** | 22.0 | 26.5*** | 25.4 | 29.2** | 21.0 | 27.9*** | 12.6 | 23.8*** |
|  | 18-19 | 14.7 | 18.4** | 18.0 | 19.9* | 19.2*** | 14.4 | 13.9 | 12.9 | 20.3*** | 14.4 | 18.9 | 22.2*** |
|  | **Age at first marriage** | |  |  |  |  |  |  |  |  |  |  |  |
|  | 20 + | 48.8*** | 10.0 | 57.5*** | 15.9 | 43.7*** | 18.4 | 46.6*** | 29.0 | 40.6*** | 20.2 | 46.8*** | 25.5 |
|  | Never married | 42.6*** | 20.0 | 34.2*** | 17.6 | 40.4*** | 23.2 | 40.1*** | 27.3 | 51.2*** | 32.9 | 41.6*** | 28.3 |
|  | < 16 years | 0.7 | 15.8*** | 0.4 | 19.7*** | 3.7 | 28.9*** | 2.5 | 12.7*** | 1.9 | 19.6*** | 1.4 | 13.6*** |
|  | 16-17 | 2.8 | 34.3*** | 2.0 | 27.6*** | 5.0 | 17.9*** | 3.8 | 15.5*** | 2.4 | 14.0*** | 3.4 | 15.8*** |
|  | 18-19 | 5.1 | 19.9*** | 5.8 | 19.2*** | 7.2 | 11.7*** | 6.9 | 15.5*** | 3.9 | 13.1*** | 6.8 | 16.8*** |
| ***HIV ⁄AIDS Awareness*** | | | | | | | | | | | | | |
|  | **HIV ⁄AIDS awareness** | |  |  |  |  |  |  |  |  |  |  |  |
|  | Low | 24.7 | 39.7*** | 35.1 | 38.2* | 25.4 | 41.5*** | 21.6 | 35.7*** | 20.1 | 30.8*** | 27.2 | 34.4*** |
|  | Average | 38.1*** | 30.5 | 44.5** | 40.3 | 37.6*** | 28.8 | 33.2 | 34.7 | 43.2* | 38.7 | 44.8 | 42.3 |
|  | High | 37.2*** | 29.8 | 20.4 | 21.5 | 37.0*** | 29.7 | 45.2*** | 29.6 | 36.7** | 30.4 | 28.0*** | 23.3 |
|  No condom use at last sexual intercourse with non-spousal partner  *: two-tailed chi-square test with p < 0.05  **: two-tailed chi-square test with p < 0.01  ***: two-tailed chi-square test with p < 0.001  †Occupation type was not collected | | | | | | | | | | | | | |

**Table A1**: Continued

|  |  | **Ethiopia**  **2005** | | **Ethiopia**  **2011** | | **Guinea**  **2005** | | **Liberia**  **2007** | | **Malawi**  **2004** | | **Malawi**  **2010** | |
| --- | --- | --- | --- | --- | --- | --- | --- | --- | --- | --- | --- | --- | --- |
|  |  | **Male** | **Female** | **Male** | **Female** | **Male** | **Female** | **Male** | **Female** | **Male** | **Female** | **Male** | **Female** |
|  | **Sample Size** | 2917 | 3271 | 3095 | 3811 | 2234 | 3020 | 3075 | 3849 | 4774 | 5969 | 6333 | 8711 |
| ***Socio-economic and Demographic Characteristics*** | | | | | | | | | | | | | |
|  | Residence |  |  |  |  |  |  |  |  |  |  |  |  |
|  | Rural | 86.2*** | 82.9 | 78.0*** | 76.1 | 61.8 | 68.5*** | 59.6 | 57.8 | 81.9 | 84.7 | 79.0 | 80.4 |
|  | Urban | 13.8 | 17.1 | 22.0 | 23.9 | 38.1 | 31.5 | 40.4 | 42.2 | 18.1 | 15.3 | 21.0 | 19.6 |
|  | **Sex of household head** | |  |  |  |  |  |  |  |  |  |  |  |
|  | Male | 87.7 | 78.4 | 87.9 | 75.8 | 91.4 | 85.8 | 80.4 | 65.0 | 91.8 | 75.7 | 85.9 | 72.5 |
|  | Female | 12.3 | 21.6 | 12.1 | 24.2*** | 8.6 | 14.2*** | 19.6 | 35.0*** | 8.2 | 24.3*** | 14.1 | 27.5*** |
|  | **Age group (in years)** | |  |  |  |  |  |  |  |  |  |  |  |
|  | 15-19 | 22.1 | 24.4* | 21.2 | 24.4*** | 21.4 | 21.0 | 19.1 | 18.3 | 18.1 | 18.6 | 24.9*** | 21.8 |
|  | 20-29 | 29.6 | 35.4*** | 32.8 | 36.8*** | 24.3 | 30.8*** | 32.2 | 35.3** | 36.9 | 42.4*** | 32.4 | 39.6*** |
|  | 30-39 | 23.5 | 24.1 | 22.5 | 24.0* | 21.4 | 28.8*** | 26.7 | 27.3 | 25.5 | 23.8 | 24.0 | 24.6 |
|  | 40 + | 24.8*** | 16.1 | 23.5*** | 14.8 | 32.9*** | 19.3 | 22.0** | 19.1 | 19.5*** | 15.2 | 18.7*** | 14.0 |
|  | **Education level** | |  |  |  |  |  |  |  |  |  |  |  |
|  | None | 43.2 | 65.3*** | 32.5 | 50.9*** | 51.3 | 76.8*** | 18.2 | 42.7*** | 11.6 | 23.7*** | 6.6 | 15.5** |
|  | Primary | 38.0-*** | 23.5 | 51.3*** | 38.2 | 16.8*** | 12.1 | 33.6 | 33.0 | 62.3 | 62.5 | 62.9 | 64.4 |
|  | Secondary and above | 18.8*** | 11.2 | 16.1*** | 10.9 | 31.9*** | 11.1 | 48.3*** | 24.0 | 26.1*** | 13.8 | 30.5*** | 20.1 |
|  | **Standard of living** | |  |  |  |  |  |  |  |  |  |  |  |
|  | 1^st^ Quintile (lowest) | 23.5 | 24.9 | 24.8 | 25.7 | 21.5 | 23.1 | 25.9 | 26.9 | 17.9 | 24.2*** | 20.4 | 24.2*** |
|  | 2^nd^ Quintile | 30.8 | 31.5 | 31.9* | 30.6 | 20.0 | 22.0** | 20.4* | 18.7 | 23.4 | 23.0 | 14.4 | 14.5 |
|  | 3^rd^ Quintile | 27.3*** | 22.8 | 13.2* | 12.1 | 12.8 | 13.4 | 11.2 | 12.2 | 32.2 | 30.5 | 24.6*** | 22.1 |
|  | 4^th^ Quintile | 9.75 | 10.1 | 18.9 | 18.9 | 20.5 | 21.5 | 28.2 | 29.9* | 3.7 | 3.1 | 17.3 | 16.8 |
|  | 5^th^ Quintile | 8.74 | 10.7*** | 11.2 | 12.7*** | 25.2*** | 20.0 | 14.3** | 12.2 | 22.8* | 19.2 | 23.3 | 22.4 |
|  | **Occupation type** | |  |  |  |  |  |  |  |  |  |  |  |
|  | Agricultural | 75.4*** | 18.6 | 70.7*** | 26.8 | 44.5 | 44.7 | 43.7 | 56.2 | 46.5 | 43.6 | 45.0*** | 40.4 |
|  | Unemployed | 12.0 | 65.2*** | 6.4 | 42.3*** | 23.5*** | 18.6 | 20.6*** | 1.2 | 21.2 | 39.6*** | 11.4 | 28.2*** |
|  | Domestic |  |  |  |  |  |  | 0.2 | 0.6* | 1.8* | 0.7 | 1.1 | 1.4 |
|  | Trade | 5.5 | 11.0*** | 8.4 | 18.3*** | 8.3 | 28.4*** | 10.1 | 36.0*** | 8.4 | 11.6*** | 9.7 | 16.6*** |
|  | Manual labor | 4.8 | 3.8 | 8.2 | 8.6 | 16.4*** | 6.9 | 11.5*** | 1.4 | 13.3*** | 2.4 | 24.5*** | 9.6 |
|  | Office / service | 0.3 | 0.4*** | 2.4 | 2.3 | 1.1*** | 0.1 | 7.6*** | 2.0 | 4.9*** | 0.8 | 5.1*** | 2.3 |
|  | Professional / manager | 2.0*** | 1.0 | 3.9*** | 1.7 | 6.2*** | 1.3 | 6.3*** | 2.6 | 3.9*** | 1.4 | 3.3*** | 1.5 |
|  | **Marital status** | |  |  |  |  |  |  |  |  |  |  |  |
|  | Married | 57.6 | 64.2*** | 57.9 | 62.5*** | 59.8 | 78.9*** | 57.5 | 64.4*** | 65.9 | 74.1 | 58.4 | 67.4 |
|  | Never married | 39.3*** | 25.3 | 39.2*** | 26.9 | 36.4*** | 16.6 | 37.2*** | 25.6 | 31.1 | 14.7 | 38.1 | 19.5 |
|  | Separated / divorced / widowed | 3.2 | 10.5*** | 2.9 | 10.6*** | 3.8 | 4.5 | 5.3 | 10.0*** | 3.0 | 11.2 | 3.5 | 13.1 |
| ***Sexual behaviors*** | | | | | | | | | | | | | |
|  | **sexual behavior risk** | | |  |  |  |  |  |  |  |  |  |  |
|  | No | 98.0 | 98.7 | 96.7 | 95.0 | 81.2 | 93.0 | 59.7 | 60.5 | 91.8 | 96.0 | 89.2 | 93.5 |
|  | Yes | 2.0 | 1.3 | 3.3 | 5.0*** | 18.8*** | 7.0 | 40.3 | 39.5 | 8.2*** | 4.0 | 10.8*** | 6.5 |
|  | **Premarital sex** | |  |  |  |  |  |  |  |  |  |  |  |
|  | No | 72.8 | 91.0 | 79.0 | 93.3 | 31.2 | 79.5 | 21.9 | 38.3 | 30.6 | 66.1 | 34.1 | 67.8 |
|  | Yes | 27.2*** | 9.0 | 21.0*** | 6.7 | 68.8*** | 20.5 | 78.1*** | 61.7 | 69.4*** | 33.9 | 65.9*** | 32.2 |
|  | **Multiple sex partners** | |  |  |  |  |  |  |  |  |  |  |  |
|  | No | 95.6 | 97.7 | 94.9 | 97.5 | 65.4 | 89.2 | 55.7 | 71.7 | 80.9 | 93.6 | 80.3 | 93.0 |
|  | Yes | 4.4*** | 2.3 | 5.1 | 2.5 | 34.6*** | 10.8 | 44.3 | 28.3 | 19.1*** | 6.4 | 19.7*** | 7.0 |
|  | **Age at first sex** | |  |  |  |  |  |  |  |  |  |  |  |
|  | 20 + | 37.7*** | 10.7 | 38.0*** | 11.7 | 32.6*** | 5.7 | 17.4*** | 4.1 | 24.0*** | 10.4 | 23.6*** | 10.8 |
|  | Never had sex | 33.6*** | 23.9 | 32.4*** | 25.2 | 12.4** | 9.5 | 10.9*** | 5.0 | 10.3 | 9.3 | 13.9 | 13.0 |
|  | < 16 years | 4.8 | 38.2*** | 6.2 | 34.7*** | 19.4 | 49.4*** | 18.3 | 44.9*** | 25.3 | 34.2*** | 30.1 | 31.3 |
|  | 16-17 | 8.4 | 15.7*** | 8.9 | 15.8 | 16.9 | 24.5*** | 28.9 | 34.8*** | 19.0 | 25.3*** | 14.7 | 25.4*** |
|  | 18-19 | 15.5*** | 11.5 | 14.5* | 12.6 | 18.7*** | 10.9 | 24.5*** | 11.2 | 21.4 | 20.9 | 17.7 | 19.5* |
|  | **Age at first marriage** | |  |  |  |  |  |  |  |  |  |  |  |
|  | 20 + | 42.8*** | 11.3 | 41.3*** | 12.3 | 51.9*** | 9.3 | 45.2*** | 21.6 | 50.6*** | 15.9 | 45.8*** | 15.8 |
|  | Never married | 40.6 | 25.3 | 39.2*** | 26.9 | 36.5*** | 16.5 | 37.2*** | 25.6 | 31.1*** | 14.7 | 38.1*** | 19.5 |
|  | < 16 years | 1.9 | 37.9*** | 3.9 | 33.7*** | 2.1 | 40.2*** | 2.3 | 22.0*** | 2.1 | 23.1*** | 2.0 | 20.2*** |
|  | 16-17 | 5.4 | 14.8*** | 5.8 | 15.1*** | 3.8 | 22.9*** | 5.9 | 17.8*** | 4.8 | 25.9*** | 4.2 | 25.5*** |
|  | 18-19 | 9.3 | 10.8 | 9.8 | 11.9*** | 5.7 | 11.1*** | 9.4 | 13.0*** | 11.5 | 20.3*** | 9.9 | 19.0*** |
| ***HIV ⁄AIDS Awareness*** | | | | | | | | | | | | | |
|  | **HIV ⁄AIDS awareness** | |  |  |  |  |  |  |  |  |  |  |  |
|  | Low | 24.6 | 41.9*** | 25.7 | 36.2*** | 23.1 | 39.8*** | 29.8 | 35.0** | 34.6 | 32.9 | 35.5* | 32.3 |
|  | Average | 36.9* | 33.7 | 35.2 | 34.2 | 35.3 | 35.1 | 35.0 | 33.7 | 32.1 | 36.8* | 35.5 | 37.1 |
|  | High | 38.5*** | 24.4 | 39.1*** | 29.6 | 41.6*** | 25.1 | 35.2* | 31.3 | 33.3 | 30.3 | 29.0 | 30.6 |
|  No condom use at last sexual intercourse with non-spousal partner  *: two-tailed chi-square test with p < 0.05  **: two-tailed chi-square test with p < 0.01  ***: two-tailed chi-square test with p < 0.001 | | | | | | | | | | | | | |

**Table A1**: Continued

|  |  | **Mali**  **2006** | | **Mozambique**  **2009** | | **Niger**  **2006** | | **D.R. Congo**  **2007** | | **Rwanda**  **2005** | | **Rwanda**  **2010** | |
| --- | --- | --- | --- | --- | --- | --- | --- | --- | --- | --- | --- | --- | --- |
|  |  | **Male** | **Female** | **Male** | **Female** | **Male** | **Female** | **Male** | **Female** | **Male** | **Female** | **Male** | **Female** |
|  | **Sample Size** | 3886 | 4743 | 4404 | 5901 | 3232 | 4441 | 4304 | 4632 | 4728 | 5663 | 6296 | 6952 |
| ***Socio-economic and Demographic Characteristics*** | | | | | | | | | | | | | |
|  | **Residence** |  |  |  |  |  |  |  |  |  |  |  |  |
|  | Rural | 63.4 | 65.6* | 65.0 | 68.6*** | 74.5 | 80.2*** | 56.8 | 54.3 | 82.6 | 83.3 | 84.2 | 84.8 |
|  | Urban | 36.6 | 34.4 | 35.0 | 31.4 | 25.5 | 19.8 | 43.2 | 45.7 | 17.4 | 16.7 | 15.8 | 15.2 |
|  | **Sex of household head** | |  |  |  |  |  |  |  |  |  |  |  |
|  | Male | 94.7 | 87.3 | 86.8 | 64.8 | 93.3 | 80.2 | 88.2 | 78.5 | 80.5 | 63.6 | 82.3 | 65.9 |
|  | Female | 5.3 | 12.7*** | 13.2 | 35.2*** | 6.7 | 19.8*** | 11.8 | 21.5*** | 19.5 | 36.4 | 17.7 | 34.1 |
|  | **Age group (in years)** | |  |  |  |  |  |  |  |  |  |  |  |
|  | 15-19 | 21.2 | 22.0 | 19.1*** | 14.9 | 17.7 | 18.4 | 20.6 | 20.0 | 22.9 | 23.4 | 22.9 | 22.1 |
|  | 20-29 | 26.1 | 35.4*** | 28.2 | 33.0*** | 27.3 | 37.5*** | 31.6 | 38.7*** | 32.7 | 34.8* | 34.7 | 38.2*** |
|  | 30-39 | 22.5 | 24.9 | 24.2 | 25.5 | 22.6 | 27.4*** | 22.0 | 24.2* | 19.6 | 23.8*** | 19.0 | 23.1*** |
|  | 40 + | 30.2*** | 17.7 | 28.5 | 26.6 | 32.4*** | 16.7 | 25.8*** | 17.1 | 24.8*** | 18.0 | 23.4*** | 16.6 |
|  | **Education level** | |  |  |  |  |  |  |  |  |  |  |  |
|  | None | 60.0 | 77.6*** | 11.5 | 32.9*** | 68.0 | 83.0*** | 6.4 | 21.8*** | 17.5 | 22.5*** | 12.0 | 15.3*** |
|  | Primary | 19.3*** | 12.2 | 64.6*** | 55.5 | 17.6*** | 10.7 | 29.6 | 39.4*** | 70.5** | 67.6 | 68.4 | 68.6 |
|  | Secondary and above | 20.7*** | 10.2 | 23.9*** | 11.6 | 14.4*** | 6.3 | 64.0*** | 38.8 | 12.1*** | 9.9 | 19.6*** | 16.1 |
|  | **Standard of living** | |  |  |  |  |  |  |  |  |  |  |  |
|  | 1^st^ Quintile (lowest) | 23.1 | 21.9 | 26.5 | 28.3* | 45. 1 | 49.0*** | 23.1 | 24.7 | 32.8 | 37.0*** | 22.8 | 26.4*** |
|  | 2^nd^ Quintile | 17.0 | 18.4* | 23.8 | 23.7 | 1.2 | 1.2 | 18.9* | 17.0 | 7.8 | 7.4 | 21.0 | 22.4* |
|  | 3^rd^ Quintile | 18.2 | 18.3 | 18.0 | 18.2 | 23.1 | 23.8 | 23.1 | 23.0 | 22.6* | 21.1 | 23.1 | 22.3 |
|  | 4^th^ Quintile | 20.0 | 20.9 | 16.0 | 16.5 | 16.7 | 15.6 | 15.5 | 14.9 | 18.0 | 17.6 | 16.2*** | 14.5 |
|  | 5^th^ Quintile | 21.7 | 20.5 | 15.7** | 13.3 | 13.9*** | 10.4 | 19.4 | 20.3 | 18.8** | 16.9 | 16.9*** | 14.4 |
|  | **Occupation type** | |  |  |  |  |  |  |  |  |  |  |  |
|  | Agricultural | 41.8*** | 29.0 | 53.4 | 81.1*** | 41.0*** | 18.3 | 35.5 | 45.1*** | 35.3 | 59.0*** | 57.9 | 64.5*** |
|  | Unemployed | 27.5 | 40.5*** |  |  | 15.0 | 53.9*** | 27.9 | 32.0* | 43.0*** | 30.9 | 7.5 | 16.7*** |
|  | Domestic | 0.1 | 2.5*** | 1.0 | 1.8 | 0.9** | 0.4 |  |  |  |  | 2.4 | 2.2 |
|  | Trade | 13.7 | 20.1*** | 13.6* | 10.8 | 15.7 | 17.1 |  |  | 3.3 | 3.4 | 4.8 | 5.0 |
|  | Manual labor | 12.4*** | 6.9 | 20.7*** | 2.5 | 15.4*** | 7.4 | 18.1*** | 3.4 | 14.4*** | 4.0 | 22.5*** | 9.1 |
|  | Office / service | 1.2*** | 0.2 | 4.2*** | 1.2 | 9.2*** | 2.2 | 8.1 | 17.7*** | 0.7 | 0.7 | 2.1*** | 0.9 |
|  | Professional / manager | 3.3*** | 0.8 | 7.1*** | 2.6 | 2.8*** | 0.7 | 10.4*** | 1.8 | 3.3*** | 2.0 | 2.8*** | 1.6 |
|  | **Marital status** | |  |  |  |  |  |  |  |  |  |  |  |
|  | Married | 64.9 | 84.5*** | 68.1 | 71.0* | 66.2 | 85.6*** | 56.9 | 66.3*** | 51.8*** | 48.1 | 52.0** | 49.9 |
|  | Never married | 31.3*** | 11.6 | 26.4*** | 10.6 | 31.6*** | 10.4 | 38.1*** | 24.2 | 45.7*** | 38.6 | 45.5*** | 39.5 |
|  | Separated / divorced / widowed | 3.8 | 3.9 | 5.5 | 18.4*** | 2.2 | 4.0** | 5.0 | 9.5*** | 2.5 | 13.2*** | 2.55 | 10.5*** |
| ***Sexual Behaviors*** | | | | | | | | | | | | | |
|  | **sexual behavior risk** | | |  |  |  |  |  |  |  |  |  |  |
|  | No | 90.4 | 93.4 | 81.2 | 86.4 | 97.4 | 99.2 | 77.2 | 82.7 | 95.2 | 96.5 | 97.4 | 93.7 |
|  | Yes | 9.6** | 6.6 | 18.8*** | 13.6 | 2.6*** | 0.8 | 22.8*** | 17.3 | 4.8** | 3.5 | 2.6 | 6.3*** |
|  | **Premarital sex** |  |  |  |  |  |  |  |  |  |  |  |  |
|  | No | 59.3 | 81.4 | 33.4 | 66.7 | 78.3 | 96.9 | 28.1 | 51.7 | 52.9 | 82.0 | 58.2 | 82.9 |
|  | Yes | 40.7*** | 18.6 | 66.6*** | 33.3 | 21.7*** | 3.1 | 71.9 | 48.3 | 47.1 | 18.0 | 41.8*** | 17.1 |
|  | **Multiple sex partners** | |  |  |  |  |  |  |  |  |  |  |  |
|  | No | 84.1 | 94.0 | 68.4 | 85.4 | 94.3 | 98.9 | 69.6 | 85.2 | 91.5 | 95.5 | 90.7 | 94.5 |
|  | Yes | 15.9*** | 6.0 | 31.6*** | 14.6 | 5.7*** | 1.1 | 30.4*** | 14.8 | 8.5*** | 4.5 | 9.3*** | 5.5 |
|  | **Age at first sex** | |  |  |  |  |  |  |  |  |  |  |  |
|  | 20 + | 42.9*** | 7.7 | 19.4*** | 12.1 | 48.4*** | 6.9 | 18.9*** | 9.7 | 34.5*** | 31.2 | 39.6*** | 35.0 |
|  | Never had sex | 22.8*** | 10.4 | 7.5*** | 3.89 | 23.5*** | 10.0 | 12.5 | 11.7 | 28.3 | 31.2** | 27.4 | 30.4*** |
|  | < 16 years | 8.87 | 50.0*** | 28.3 | 41.4*** | 6.0 | 55.1*** | 29.5 | 36.4*** | 12.4*** | 7.6 | 11.2*** | 6.7 |
|  | 16-17 | 12.01 | 21.7*** | 23.9 | 27.4** | 9.0 | 19.7*** | 20.1 | 25.3** | 9.6 | 12.0.*** | 8.0 | 11.6*** |
|  | 18-19 | 13.41** | 10.2 | 20.9*** | 15.2 | 13.1*** | 8.3 | 19.0 | 16.9 | 15.2 | 17.8*** | 13.8 | 16.3*** |
|  | **Age at first marriage** | |  |  |  |  |  |  |  |  |  |  |  |
|  | 20 + | 54.5*** | 12.2 | 46.6*** | 20.8 | 50.8*** | 7.2 | 46.8*** | 22.2 | 45.7*** | 32.0 | 46.5*** | 35.2 |
|  | Never married | 31.4*** | 11.6 | 26.7*** | 10.7 | 31.6*** | 10.4 | 38.2*** | 24.2 | 45.8*** | 38.6 | 45.5*** | 39.5 |
|  | < 16 years | 2.0 | 44.0*** | 6.3 | 29.9*** | 1.6 | 56.3*** | 2.2 | 18.4*** | 0.4 | 3.8*** | 0.3 | 3.3*** |
|  | 16-17 | 5.2 | 20.9*** | 7.9 | 21.7*** | 5.3 | 18.4*** | 4.1 | 19.6*** | 2.1 | 9.9*** | 2.0 | 8.5*** |
|  | 18-19 | 6.9 | 11.3*** | 12.7 | 16.9*** | 10.7** | 7.7 | 8.6 | 15.6*** | 6.0 | 15.7*** | 5.7 | 13.5*** |
| ***HIV ⁄AIDS Awareness*** | | | | | | | | | | | | | |
|  | **HIV ⁄AIDS awareness** | |  |  |  |  |  |  |  |  |  |  |  |
|  | Low | 25.5 | 36.3*** | 38.2 | 36.0 | 26.4 | 44.3*** | 40.8 | 40.9 | 30.4 | 38.2*** | 39.0*** | 34.5 |
|  | Average | 36.6 | 31.5 | 32.8 | 33.0 | 40.0 | 36.7 | 27.4 | 26.5 | 38.1*** | 31.2 | 34.1* | 32.1 |
|  | High | 37.9** | 32.2 | 29.0 | 31.0 | 33.6*** | 19.0 | 31.8 | 32.6 | 31.5 | 30.5 | 26.9 | 33.4*** |
|  No condom use at last sexual intercourse with non-spousal partner  *: two-tailed chi-square test with p < 0.05  **: two-tailed chi-square test with p < 0.01  ***: two-tailed chi-square test with p < 0.001 | | | | | | | | | | | | | |

**Table A1**: Continued

|  |  | **Sao Tome & Principe 2008/09** | | **Senegal**  **2005** | | **Senegal**  **2011** | | **Sierra Leone**  **2008** | | **Swaziland**  **2006/07** | | **Uganda**  **2011** | |
| --- | --- | --- | --- | --- | --- | --- | --- | --- | --- | --- | --- | --- | --- |
|  |  | **Male** | **Female** | **Male** | **Female** | **Male** | **Female** | **Male** | **Female** | **Male** | **Female** | **Male** | **Female** |
|  | **Sample Size** | 2160 | 2550 | 3250 | 4466 | 4327 | 5590 | 3009 | 3466 | 3602 | 4584 | 9399 | 12000 |
| ***Socio-economic and Demographic Characteristics*** | | | | | | | | | | | | | |
|  | **Residence** |  |  |  |  |  |  |  |  |  |  |  |  |
|  | Rural | 50.5** | 45.2 | 43.7 | 48.4*** | 44.8 | 51.0*** | 62.7 | 65.0* | 71.6 | 73.5 | 81.0** | 79.4 |
|  | Urban | 49.5 | 54.8 | 56.3 | 51.6 | 55.2 | 49.0 | 37.3 | 35.0 | 28.4 | 26.5 | 19.0 | 20.6 |
|  | **Sex of household head** | |  |  |  |  |  |  |  |  |  |  |  |
|  | Male | 80.4 | 58.2 | 82.2 | 74.1 | 82.2 | 75.3 | 85.6 | 76.9 | 64.5 | 42.5 | 83.9 | 61.3 |
|  | Female | 19.6 | 41.8*** | 17.8 | 25.9*** | 17.8 | 24.7*** | 14.4 | 23.1*** | 35.5 | 57.5*** | 16.1 | 38.7*** |
|  | **Age group (in years)** | |  |  |  |  |  |  |  |  |  |  |  |
|  | 15-19 | 24.7* | 20.9 | 26.0 | 25.0 | 24.1 | 22.6 | 16.1 | 15.4 | 33.8*** | 26.0 | 21.6* | 20.2 |
|  | 20-29 | 27.6 | 35.1*** | 31.0 | 35.8*** | 32.2 | 37.0*** | 25.5 | 38.6*** | 35.4 | 35.5 | 28.8 | 33.8*** |
|  | 30-39 | 23.0 | 25.3 | 19.0 | 23.4** | 19.8 | 24.6*** | 28.1 | 30.1 | 18.6 | 22.1** | 23.8 | 23.4 |
|  | 40 + | 24.7*** | 18.8 | 24.0*** | 15.8 | 23.9*** | 15.8 | 30.3*** | 15.9 | 12.2 | 16.4*** | 25.8*** | 22.6 |
|  | **Education level** | |  |  |  |  |  |  |  |  |  |  |  |
|  | None | 1.4 | 5.8*** | 41.9 | 56.7*** | 38.8 | 57.5*** | 50.0 | 67.9*** | 7.6 | 8.2 | 6.3 | 16.2*** |
|  | Primary | 56.8 | 57.9 | 26.6 | 25.4 | 28.0*** | 21.5 | 13.8 | 12.3 | 35.5 | 33.5 | 57.2 | 58.5 |
|  | Secondary and above | 41.8** | 36.3 | 31.5*** | 17.9 | 33.2*** | 21.0 | 36.2*** | 19.8 | 56.9 | 58.3 | 36.6*** | 25.3 |
|  | **Standard of living** | |  |  |  |  |  |  |  |  |  |  |  |
|  | 1^st^ Quintile (lowest) | 18.0 | 17.4 | 15.5 | 15.0 | 12.7 | 12.7 | 28.0 | 28.3 | 23.0 | 25.4* | 27.0 | 27.1 |
|  | 2^nd^ Quintile | 19.1 | 17.3 | 18.7 | 18.2 | 14.7 | 15.8 | 17.6 | 19.2 | 16.3 | 15.5 | 14.1*** | 12.8 |
|  | 3^rd^ Quintile | 19.8 | 18.6 | 16.0 | 17.8 | 18.6 | 19.8 | 17.7 | 18.4 | 20.7 | 19.7 | 18.2 | 18.4 |
|  | 4^th^ Quintile | 22.2 | 23.5 | 27.9 | 26.4 | 24.4 | 23.2 | 18.5 | 18.1 | 22.4 | 21.7 | 21.8 | 22.7 |
|  | 5^th^ Quintile | 20.9 | 23.2 | 21.9 | 22.6 | 29.6 | 28.5 | 18.2* | 16.0 | 17.6 | 17.7 | 18.9 | 19.0 |
|  | **Occupation type** | |  |  |  |  |  |  |  |  |  |  |  |
|  | Agricultural | 25.2*** | 7.3 | 23.4*** | 10.9 | 25.1*** | 9.0 | 52.1*** | 43.8 | 9.6*** | 4.0 | 26.7*** | 13.3 |
|  | Unemployed | 14.1 | 43.1*** | 24.8 | 55.1*** | 16.0 | 53.8*** | 14.2 | 23.1*** | 45.6 | 56.9*** | 16.3 | 29.5*** |
|  | Domestic | -- | -- | -- | -- | 0.2 | 4.6*** | 0.1 | 0.1 | -- | -- | 0.5 | 1.3*** |
|  | Trade | 2.0 | 24.9*** | 11.9 | 17.4*** | 12.7 | 19.1*** | 8.7 | 26.2*** | 5.2 | 9.7*** | 6.8 | 10.7*** |
|  | Manual labor | 17.6*** | 7.4 | 28.9*** | 3.6 | 30.3*** | 1.6 | 9.6*** | 1.2 | 20.5*** | 6.7 | 36.0*** | 29.7 |
|  | Office / service | 37.4*** | 14.7 | 3.2 | 10.3*** | 13.9* | 11.6 | 3.8*** | 1.5 | 12.3 | 18.0*** | 6.4 | 12.2*** |
|  | Professional / manager | 3.7* | 2.6 | 7.8*** | 2.7 | 1.9*** | 0.3 | 11.5*** | 4.1 | 6.8*** | 4.7 | 7.0*** | 3.3 |
|  | **Marital status** | |  |  |  |  |  |  |  |  |  |  |  |
|  | Married | 50.0 | 66.3*** | 48.0 | 65.3*** | 42.1 | 66.3*** | 63.8 | 76.7*** | 28.1 | 40.9*** | 60.0 | 62.7*** |
|  | Never married | 40.5*** | 22.9 | 48.7*** | 29.2 | 56.2*** | 29.3 | 33.0*** | 17.0 | 67.0*** | 50.0 | 33.6*** | 22.0 |
|  | Separated / divorced / widowed | 9.5 | 10.8 | 3.3 | 5.5** | 1.7 | 4.4*** | 3.2 | 6.3*** | 4.9 | 9.0*** | 6.4 | 15.3*** |
| ***Sexual Behaviors*** | | | | | | | | | | | | | |
|  | **sexual behavior risk** | | |  |  |  |  |  |  |  |  |  |  |
|  | No | 64.6 | 47.4 | 91.9 | 97.4 | 94.7 | 97.7 | 75.4 | 84.8 | 85.5 | 80.1 | 87.4 | 87.2 |
|  | Yes | 35.4 | 52.6*** | 8.1*** | 2.6 | 5.3*** | 2.3 | 24.6*** | 15.2 | 14.5 | 19.9*** | 12.6 | 12.8 |
|  | **Premarital sex** | |  |  |  |  |  |  |  |  |  |  |  |
|  | No | 25.9 | 53.8 | 50.1 | 91.6 | 54.5 | 86.0 | 28.3 | 60.6 | 37.8 | 31.4 | 34.0 | 54.5 |
|  | Yes | 74.1*** | 46.2 | 49.9*** | 8.4 | 45.5*** | 14.0 | 71.7*** | 39.4 | 62.2 | 68.6*** | 66.0*** | 45.5 |
|  | **Multiple sex partners** | |  |  |  |  |  |  |  |  |  |  |  |
|  | No | 65.5 | 89.3 | 76.5 | 95.6 | 83.8 | 96.8 | 66.7 | 84.8 | 65.2 | 69.1 | 76.1 | 88.2 |
|  | Yes | 34.5*** | 10.7 | 23.5*** | 4.4 | 16.2*** | 3.2 | 33.3*** | 15.2 | 34.8*** | 30.9 | 23.9*** | 11.8 |
|  | **Age at first sex** | |  |  |  |  |  |  |  |  |  |  |  |
|  | 20 + | 15.5 | 12.9 | 31.6*** | 18.0 | 35.9*** | 21.8 | 28.9*** | 8.1 | 22.7*** | 13.7 | 22.2*** | 10.4 |
|  | Never had sex | 15.5 | 13.1 | 28.0 | 28.8 | 34.6*** | 26.2 | 11.7*** | 5.3 | 31.7*** | 17.7 | 16.3*** | 11.8 |
|  | < 16 years | 22.7 | 19.9 | 17.2 | 24.0*** | 9.3 | 24.9*** | 19.8 | 50.7*** | 10.1 | 19.3*** | 17.8 | 31.1*** |
|  | 16-17 | 25.7 | 30.6* | 11.6 | 15.6** | 8.5 | 13.4*** | 18.3 | 21.6* | 16.6 | 26.9*** | 21.4 | 27.5*** |
|  | 18-19 | 20.6 | 23.5 | 11.6 | 13.6* | 11.7 | 13.7* | 21.3*** | 14.3 | 18.9 | 22.4*** | 22.3*** | 19.2 |
|  | **Age at first marriage** | |  |  |  |  |  |  |  |  |  |  |  |
|  | 20 + | 46.0*** | 20.9 | 41.8*** | 19.7 | 39.9*** | 24.0 | 51.3*** | 18.4 | 29.3 | 27.5 | 48.5*** | 20.6 |
|  | Never married | 40.5*** | 22.9 | 48.8*** | 29.2 | 56.2*** | 29.2 | 33.0*** | 17.0 | 67.0*** | 50.0 | 33.6*** | 22.0 |
|  | < 16 years | 0.5 | 11.7*** | 2.7 | 23.4*** | 0.6 | 21.7*** | 2.4 | 36.3*** | 0.5 | 4.5*** | 3.3 | 21.6*** |
|  | 16-17 | 3.9 | 21.8*** | 3.3 | 15.6*** | 0.9 | 12.4*** | 4.8 | 16.3*** | 0.8 | 7.7*** | 4.7 | 19.4*** |
|  | 18-19 | 9.0 | 22.7*** | 3.4 | 12.1*** | 2.4 | 12.7*** | 8.5 | 12.0*** | 2.4 | 10.3*** | 9.9 | 16.4*** |
| ***HIV ⁄AIDS Awareness*** | | | | | | | | | | | | | |
|  | **HIV ⁄AIDS awareness** | |  |  |  |  |  |  |  |  |  |  |  |
|  | Low | 32.6 | 33.4 | 33.3 | 31.9 | 28.9 | 33.9** | 26.0 | 43.1*** | 36.2** | 31.6 | 32.6 | 33.7 |
|  | Average | 35.4 | 35.2 | 34.6 | 34.3 | 32.5 | 30.7 | 34.6* | 29.8 | 40.2*** | 34.4 | 43.0 | 42.8 |
|  | High | 32.0 | 31.4 | 32.1 | 33.8 | 38.6* | 35.4 | 39.4*** | 27.1 | 23.6 | 34.0*** | 24.4 | 23.5 |
|  No condom use at last sexual intercourse with non-spousal partner  *: two-tailed chi-square test with p < 0.05  **: two-tailed chi-square test with p < 0.01  ***: two-tailed chi-square test with p < 0.001 | | | | | | | | | | | | | |

**Table A1**: Continued

|  |  | **Zambia**  **2007** | | **Zimbabwe**  **2005/06** | | **Zimbabwe**  **2010/11** | | **Cameroon**  **2011** | |
| --- | --- | --- | --- | --- | --- | --- | --- | --- | --- |
|  |  | **Male** | **Female** | **Male** | **Female** | **Male** | **Female** | **Male** | **Female** |
|  | **Sample Size** | 5161 | 5713 | 5555 | 7494 | 6045 | 7852 | 6945 | 7253 |
| ***Socio-economic and Demographic Characteristics*** | | | | | | | | | |
|  | **Residence** |  |  |  |  |  |  |  |  |
|  | Rural | 56.8 | 57.9 | 60.3 | 61.6 | 70.1 | 68.6 | 45.0 | 46.3 |
|  | Urban | 43.2 | 42.1 | 39.7 | 38.4 | 29.9 | 31.4 | 55.0 | 53.7 |
|  | **Sex of household head** | |  |  |  |  |  |  |  |
|  | Male | 88.5 | 75.9 | 76.8 | 60.7 | 71.0 | 51.2 | 86.9 | 72.6 |
|  | Female | 11.5 | 24.1*** | 23.2 | 39.3*** | 29.0 | 48.8*** | 13.1 | 27.4*** |
|  | **Age group (in years)** | |  |  |  |  |  |  |  |
|  | 15-19 | 21.6 | 21.9 | 27.7*** | 24.2 | 23.8** | 21.3 | 22.2 | 22.8 |
|  | 20-29 | 30.9 | 37.8*** | 35.3 | 38.4** | 34.7 | 38.5*** | 32.2 | 37.7*** |
|  | 30-39 | 26.0 | 25.6 | 20.7 | 23.2** | 23.7 | 25.3** | 21.4 | 23.7** |
|  | 40 + | 21.5*** | 14.8 | 16.3** | 14.2 | 17.8*** | 14.9 | 24.2*** | 15.8 |
|  | **Education level** | |  |  |  |  |  |  |  |
|  | None | 4.4 | 10.0*** | 1.2 | 4.3*** | 1.0 | 2.3*** | 9.4 | 20.8*** |
|  | Primary | 45.6 | 53.4*** | 28.0 | 32.6*** | 24.4 | 29.5*** | 33.2 | 33.1 |
|  | Secondary and above | 50.0*** | 36.6 | 70.8*** | 63.1 | 74.6*** | 68.2 | 57.4*** | 46.1 |
|  | **Standard of living** | |  |  |  |  |  |  |  |
|  | 1^st^ Quintile (lowest) | 26.4 | 29.8*** | 16.6 | 18.9* | 19.0 | 20.8** | 19.2 | 20.5* |
|  | 2^nd^ Quintile | 11.8 | 11.3 | 17.3 | 17.7 | 19.1 | 19.7 | 18.2 | 18.9 |
|  | 3^rd^ Quintile | 20.1** | 18.2 | 21.0 | 19.8 | 21.6 | 20.7 | 18.4 | 18.9 |
|  | 4^th^ Quintile | 20.1 | 19.3 | 23.5*** | 20.0 | 22.3*** | 18.9 | 23.8** | 21.7 |
|  | 5^th^ Quintile | 21.6 | 21.4 | 21.6 | 23.6* | 18.0 | 19.9** | 20.4 | 20.0 |
|  | **Occupation type** | |  |  |  |  |  |  |  |
|  | Agricultural | 40.8*** | 26.6 | 25.5*** | 15.2 | 23.0*** | 10.1 | 33.8*** | 25.8 |
|  | Unemployed | 19.8 | 46.0*** | 30.5 | 57.3*** | 31.9 | 58.3*** | 13.3 | 32.8*** |
|  | Domestic | -- | -- | 5.7 | 5.1 | 2.0 | 3.7*** | 3.1* | 2.2 |
|  | Trade | 12.8 | 17.4*** | 4.3 | 10.1*** | 6.5 | 14.9*** | 11.9 | 22.1*** |
|  | Manual labor | 16.4*** | 3.5 | 20.6*** | 4.7 | 29.2*** | 8.2 | 28.9*** | 12.7 |
|  | Office / service | 4.9** | 3.2 | 8.1*** | 4.7 | 2.3*** | 1.3 | 4.3*** | 2.9 |
|  | Professional / manager | 5.3*** | 3.3 | 5.3*** | 2.9 | 5.1*** | 3.5 | 4.7*** | 1.5 |
|  | **Marital status** | |  |  |  |  |  |  |  |
|  | Married | 55.8 | 61.4*** | 46.4 | 58.0*** | 52.1 | 62.5*** | 50.0 | 62.8*** |
|  | Never married | 39.2*** | 25.8 | 48.8*** | 26.6 | 43.3*** | 23.2 | 45.2*** | 28.3 |
|  | Separated / divorced / widowed | 5.0 | 12.8*** | 4.86 | 15.5*** | 4.6 | 14.3*** | 4.8 | 8.9*** |
| ***Sexual Behaviors*** | | | | | | | | | |
|  | **sexual behavior risk** | | |  |  |  |  |  |  |
|  | No | 88.9 | 91.8 | 94.2 | 94.9 | 95.4 | 95.5 | 83.8 | 83.6 |
|  | Yes | 11.1*** | 8.2 | 5.8 | 5.1 | 4.6 | 4.5 | 16.2 | 16.4 |
|  | **Premarital sex** |  |  |  |  |  |  |  |  |
|  | No | 27.3 | 58.9 | 41.7 | 70.5 | 43.9 | 66.3 | 32.5 | 58.7 |
|  | Yes | 72.7*** | 41.1 | 58.3*** | 29.5 | 56.1*** | 33.7 | 67.5*** | 41.3 |
|  | **Multiple sex partners** |  |  |  |  |  |  |  |  |
|  | No | 72.7 | 87.2 | 78.0 | 92.3 | 77.6 | 91.8 | 58.3 | 78.9 |
|  | Yes | 27.3*** | 12.8 | 22.0*** | 7.7 | 22.4*** | 8.2 | 41.7*** | 21.1 |
|  | **Age at first sex** | |  |  |  |  |  |  |  |
|  | 20 + | 19.9*** | 11.4 | 30.8*** | 19.9 | 34.6*** | 22.7 | 22.6*** | 9.2 |
|  | Never had sex | 14.1 | 13.0 | 25.9*** | 20.4 | 24.3*** | 17.5 | 17.4*** | 13.5 |
|  | < 16 years | 30.2 | 32.3* | 9.4 | 15.4*** | 8.1 | 13.5*** | 19.7 | 34.2*** |
|  | 16-17 | 18.3 | 26.3*** | 14.1 | 21.9*** | 13.8 | 24.0*** | 20.8 | 26.3*** |
|  | 18-19 | 17.5 | 16.9 | 19.8** | 22.4 | 19.2 | 22.3*** | 19.5*** | 16.7 |
|  | **Age at first marriage** | |  |  |  |  |  |  |  |
|  | 20 + | 48.0*** | 18.4 | 42.7*** | 23.7 | 48.0*** | 27.6 | 44.4*** | 20.6 |
|  | Never married | 39.2*** | 25.8 | 48.8*** | 26.6 | 43.3*** | 23.2 | 45.2*** | 28.3 |
|  | < 16 years | 1.1 | 17.3*** | 0.7 | 11.0*** | 0.8 | 10.7*** | 1.6 | 21.6*** |
|  | 16-17 | 3.0 | 22.9*** | 2.2 | 19.0*** | 2.1 | 18.9*** | 3.4 | 16.2*** |
|  | 18-19 | 8.7 | 15.6*** | 5.6 | 19.7*** | 5.8 | 19.6*** | 5.4 | 13.3*** |
| ***HIV ⁄AIDS Awareness*** | | | | | | | | | |
|  | **HIV ⁄AIDS awareness** | |  |  |  |  |  |  |  |
|  | Low | 38.5 | 32.1 | 34.6 | 36.8 | 27.6*** | 22.5 | 30.6 | 35.9*** |
|  | Average | 39.3 | 38.5 | 34.6* | 30.4 | 27.5*** | 23.0 | 38.2*** | 33.0 |
|  | High | 22.2 | 29.4 | 30.8 | 32.8 | 44.9 | 54.5*** | 31.2 | 31.1 |
|  No condom use at last sexual intercourse with non-spousal partner  *: two-tailed chi-square test with p < 0.05  **: two-tailed chi-square test with p < 0.01  ***: two-tailed chi-square test with p < 0.001 | | | | | | | | | |

**Table A2:** Results from Blinder-Oaxaca decomposition analysis of gender inequalities in HIV/AIDS prevalence, using earlier DHSs. conducted between 2004 and 2006, for countries surveyed twice between 2003 and 2012

| **Countries** | **Survey year** | **Gender Inequality in HIV/AIDS Prevalence**  **(women-men)** | **Composition effect^c^** | | | **Response effect^d^** | | |
| --- | --- | --- | --- | --- | --- | --- | --- | --- |
|  |  |  | **Beta**  **(SE)** | **p-value** | **Percent^a^** | **Beta**  **(SE)** | **p-value** | **Percent^b^** |
| Cameroon† | 2004 | 2.71 | -0.022 (0.030) | 0.461 | -30.0 | 0.097 (0.031) | 0.002 | 130.0 |
| Ethiopia‡ | 2005 | 0.95 | 0.007 (0.003) | 0.022 | 73.5 | 0.003 (0.004) | 0.47 | 26.5 |
| Malawi† | 2004 | 3.09 | -0.023 (0.014) | 0.099 | -72.1 | 0.055 (0.018) | 0.002 | 172.1 |
| Rwanda† | 2005 | 1.41 | 0.002 (0.004) | 0.511 | 16.6 | 0.012 (0.005) | 0.014 | 83.4 |
| Senegal | 2005 | 0.44 | 0.001 (0.003) | 0.818 | 14.8 | 0.004 (0.003) | 0.244 | 85.2 |
| Zimbabwe* | 2005/06 | 6.37 | 0.02 (0.009) | 0.024 | 30.8 | 0.045 (0.011) | 0.000 | 69.2 |
| *Note*: using this method, the net percent contribution of both components always equals to 100. A contribution may be negative (less than zero) or positive and can even exceed 100. A positive contribution indicates that the component contributes to the greater prevalence of HIV/AIDS among women relative to men, whereas a negative contribution indicates the opposite. Here, Males are the base group.  † Countries where difference between men and women in response to factors mainly explains the gap at p-value= 5%.  ‡ Countries where difference in factors distribution between men and women mainly explains the gap at p-value= 5%  * Countries where difference in both response to factors and factors distribution between men and women explains the gap at p=5%.  ^a^ Part of gender inequality in HIV/AIDS prevalence attributable to differences in the distribution of risk factors.  ^b^ Part of gender inequality in HIV/AIDS prevalence attributable to differences in the effects of risk factors.  SE = Standard Error.  ^c^ Represent the contribution to gender inequalities in HIV/AIDS prevalence due to gender differences in the distributions of observable HIV/AIDS risk factors between women and men.  ^d^ Reflect the contribution to gender inequalities in HIV/AIDS due to gender differences in the effects of measured HIV/AIDS risk factors, as well as unmeasured factors not included in the model. | | | | | | | | |

**Table A3**: Results from Blinder-Oaxaca decomposition analysis of gender inequalities in HIV/AIDS prevalence using earlier DHSs. conducted between 2004 and 2006, for countries surveyed twice between 2003 and 2012

|  |  |  | **Cameroon**  **2004** | **Ethiopia^a^**  **2005** | **Malawi**  **2004** | **Rwanda^a^ 2005** | **Senegal^a^ 2005** | **Zimbabwe 2005/06** |
| --- | --- | --- | --- | --- | --- | --- | --- | --- |
|  |  |  | **Absolute contribution to gender inequalities in HIV/AIDS prevalence (P-value)** | | | | | |
| **Gender Difference in HIV/AIDS Prevalence (%)** | | | **2.71** | **0.95** | **3.09** | **1.41** | **0.44** | **6.37** |
|  | |  |  |  |  |  |  |  |
| ***The composition effect^b^*** | | | **-0.81 (0.461)** | **0.7 (0.022)** | **-2.23 (0.099)** | **0.23 (0.511)** | **0.07 (0.818)** | **1.96 (0.024)** |
|  |  | |  |  |  |  |  |  |
|  | **Socio-economic and demographic characteristics** | | **-0.380** | **0.350** | **1.290** | **-0.270** | **0.100** | **2.630** |
|  |  | **Residence** | **0.020** | **0.060** | **-0.180** | **0.020** | **0.000** | **-0.020** |
|  |  | Urban | 0.01 (0.506) | 0.03 (0.036) | -0.09 (0.225) | 0.01 (0.686) | 0 (0.767) | -0.01 (0.211) |
|  |  | Rural | 0.01 (0.506) | 0.03 (0.036) | -0.09 (0.225) | 0.01 (0.686) | 0 (0.767) | -0.01 (0.211) |
|  |  | **Sex of household head** | **-0.140** | **0.040** | **0.740** | **-0.100** | **0.000** | **0.300** |
|  |  | Male | -0.07 (0.476) | 0.02 (0.311) | 0.37 (0.311) | -0.05 (0.745) | 0 (0.804) | 0.15 (0.152) |
|  |  | Female | -0.07 (0.476) | 0.02 (0.311) | 0.37 (0.311) | -0.05 (0.745) | 0 (0.804) | 0.15 (0.152) |
|  |  | **Age group** | **0.020** | **0.010** | **-0.230** | **-0.010** | **-0.010** | **0.790** |
|  |  | 15-19 | -0.05 (0.415) | 0 (0.6) | -0.09 (0.022) | 0 (0.674) | 0 (0.774) | 0.44 (0) |
|  |  | 20-29 | 0.02 (0.417) | 0 (0.829) | 0.25 (0.124) | 0 (0.901) | 0 (0.778) | 0.09 (0.007) |
|  |  | 30-39 | 0.05 (0.561) | 0 (0.21) | -0.14 (0.028) | -0.08 (0.681) | 0 (0.887) | 0.25 (0) |
|  |  | 40 + | 0 (0.964) | 0.01 (0.707) | -0.25 (0.096) | 0.07 (0.656) | -0.01 (0.773) | 0.01 (0.614) |
|  |  | **Education level** | **-0.180** | **-0.160** | **0.370** | **0.030** | **0.010** | **-0.150** |
|  |  | None | -0.01 (0.593) | -0.1 (0.101) | 0.14 (0.675) | 0.02 (0.696) | 0 (0.871) | -0.07 (0.303) |
|  |  | Primary | -0.14 (0.487) | -0.04 (0.267) | 0 (0.698) | 0.01 (0.691) | 0 (0.784) | 0.04 (0.449) |
|  |  | Secondary and above | -0.03 (0.765) | -0.02 (0.268) | 0.23 (0.567) | 0 (0.776) | 0.01 (0.786) | -0.12 (0.313) |
|  |  | **Standard of living** | **-0.100** | **-0.010** | **-0.710** | **-0.010** | **0.000** | **-0.250** |
|  |  | 1^st^ Quintile | -0.05 (0.728) | -0.01 (0.365) | -0.57 (0.057) | -0.01 (0.78) | 0 (0.77) | -0.08 (0.011) |
|  |  | 2^nd^ Quintile | 0 (0.998) | 0 (0.384) | 0.01 (0.373) | 0 (0.706) | 0 (0.789) | 0 (0.175) |
|  |  | 3^rd^ Quintile | -0.01 (0.672) | -0.01 (0.566) | -0.02 (0.578) | 0 (0.966) | 0 (0.776) | -0.03 (0.01) |
|  |  | 4^th^ Quintile | 0.01 (0.681) | 0 (0.319) | -0.05 (0.14) | 0 (0.69) | 0 (0.766) | -0.08 (0.044) |
|  |  | 5^th^ Quintile | -0.05 (0.774) | 0.01 (0.301) | -0.08 (0.546) | 0 (0.977) | 0 (0.79) | -0.06 (0.039) |
|  |  | **Occupation type** | **0.020** | **0.270** | **-2.510** | **0.190** | **0.070** | **0.430** |
|  |  | Unemployed | 0.8 (0.378) | 0.31 (0.085) | -1.74 (0.054) | -0.12 (0.673) | 0.01 (0.829) | 0.26 (0.348) |
|  |  | Agricultural | -0.12 (0.377) | -0.09 (0.812) | 0.39 (0.06) | 0.37 (0.664) | -0.01 (0.771) | -0.01 (0.941) |
|  |  | Domestic | 0.03 (0.378) | - | -0.06 (0.447) | - | - | -0.02 (0.101) |
|  |  | Trade | 0 (0.395) | 0.04 (0.051) | -0.05 (0.664) | 0 (0.692) | 0.01 (0.782) | 0.09 (0.293) |
|  |  | Manual labor | -0.63 (0.399) | 0 (0.82) | -0.4 (0.476) | -0.07 (0.743) | 0.04 (0.8) | -0.07 (0.821) |
|  |  | Office / service | 0.01 (0.886) | 0 (0.637) | -0.71 (0.103) | 0 (0.69) | 0.01 (0.79) | 0.13 (0.093) |
|  |  | Professional / manager | -0.07 (0.423) | 0.01 (0.166) | 0.06 (0.717) | 0.01 (0.696) | 0.01 (0.777) | 0.05 (0.444) |
|  |  | **Marital status** | **-0.020** | **0.140** | **3.810** | **-0.390** | **0.030** | **1.530** |
|  |  | Never married | 0.02 (0.366) | 0.09 (0.131) | 2.55 (0.119) | -0.07 (0.696) | 0.03 (0.774) | 1.07 (0.002) |
|  |  | Married | -0.02 (0.39) | 0 (0.895) | -0.04 (0.878) | -0.05 (0.68) | 0 (0.798) | -0.6 (0) |
|  |  | Separated / divorced / widowed | -0.02 (0.455) | 0.05 (0.057) | 1.3 (0.046) | -0.27 (0.682) | 0 (0.772) | 1.06 (0) |
|  | **Sexual Behavior Factors** | | **-0.440** | **0.330** | **-3.500** | **0.450** | **-0.050** | **-0.640** |
|  |  | **sexual behavior risk** | **-0.020** | **0.000** | **0.440** | **-0.020** | **0.000** | **-0.020** |
|  |  | No | -0.01 (0.505) | 0 (0.435) | 0.22 (0.242) | -0.01 (0.699) | 0 (0.751) | -0.01 (0.153) |
|  |  | Yes | -0.01 (0.505) | 0 (0.435) | 0.22 (0.242) | -0.01 (0.699) | 0 (0.751) | -0.01 (0.153) |
|  |  | **Premarital sex** | **0.000** | **-0.080** | **-3.340** | **0.380** | **0.000** | **-1.860** |
|  |  | No | 0 (0.802) | -0.04 (0.143) | -1.67 (0.018) | 0.19 (0.659) | 0 (0.836) | -0.93 (0) |
|  |  | Yes | 0 (0.802) | -0.04 (0.143) | -1.67 (0.018) | 0.19 (0.659) | 0 (0.836) | -0.93 (0) |
|  |  | **Multiple sex partners** | **-0.060** | **-0.020** | **-1.620** | **0.040** | **-0.060** | **-0.240** |
|  |  | No | -0.03 (0.563) | -0.01 (0.048) | -0.81 (0.145) | 0.02 (0.701) | -0.03 (0.762) | -0.12 (0.377) |
|  |  | Yes | -0.03 (0.563) | -0.01 (0.048) | -0.81 (0.145) | 0.02 (0.701) | -0.03 (0.762) | -0.12 (0.377) |
|  |  | **Age at first sex** | **-0.360** | **0.430** | **1.020** | **0.050** | **0.010** | **1.480** |
|  |  | Never had sex | -0.41 (0.359) | 0.22 (0.031) | 0.02 (0.838) | 0.05 (0.692) | 0 (0.766) | 0.96 (0) |
|  |  | < 16 years | 0.1 (0.398) | 0.21 (0.076) | 0.43 (0.266) | 0.02 (0.731) | 0 (0.797) | 0.35 (0) |
|  |  | 16-17 | 0.01 (0.449) | 0.07 (0.04) | 0.12 (0.608) | -0.01 (0.715) | 0 (0.767) | 0.38 (0) |
|  |  | 18-19 | -0.12 (0.361) | -0.02 (0.127) | 0.01 (0.717) | -0.02 (0.697) | 0 (0.788) | 0.11 (0) |
|  |  | 20 + | 0.06 (0.764) | -0.05 (0.566) | 0.44 (0.509) | 0.01 (0.733) | 0.01 (0.782) | -0.32 (0.014) |
|  | **HIV ⁄AIDS Awareness** | | **0.000** | **0.010** | **-0.030** | **0.060** | **0.000** | **-0.030** |
|  |  | **HIV ⁄AIDS awareness** | **0.000** | **0.010** | **-0.030** | **0.060** | **0.000** | **-0.030** |
|  |  | low | -0.01 (0.785) | 0 (0.916) | 0.01 (0.792) | 0.05 (0.682) | 0 (0.81) | -0.01 (0.762) |
|  |  | average | 0.01 (0.706) | 0 (0.689) | -0.01 (0.884) | 0.01 (0.784) | 0 (0.808) | -0.02 (0.566) |
|  |  | high | 0 (0.596) | 0.01 (0.632) | -0.03 (0.671) | 0 (0.687) | 0 (0.794) | 0 (0.802) |
|  |  |  |  |  |  |  |  |  |
| ***The response effect^c^*** | | | **3.52 (0.002)** | **0.25 (0.47)** | **5.32 (0.002)** | **1.18 (0.014)** | **0.37 (0.244)** | **4.41 (0)** |
|  |  | |  |  |  |  |  |  |
|  | **Socio-economic and demographic characteristics** | | **1.540** | **0.670** | **-1.380** | **-0.730** | **0.030** | **2.440** |
|  |  | **Residence** | **0.160** | **0.480** | **0.020** | **0.080** | **0.000** | **0.030** |
|  |  | Urban | 0.19 (0.41) | -0.09 (0.722) | -0.01 (0.965) | -0.02 (0.558) | 0.01 (0.408) | -0.05 (0.846) |
|  |  | Rural | -0.03 (0.41) | 0.57 (0.722) | 0.03 (0.965) | 0.1 (0.558) | -0.01 (0.408) | 0.08 (0.846) |
|  |  | **Sex of household head** | **0.120** | **0.120** | **0.590** | **-0.310** | **0.000** | **-0.140** |
|  |  | Male | 0.16 (0.503) | 0.14 (0.746) | 0.65 (0.537) | -0.41 (0.146) | 0 (0.81) | -0.2 (0.696) |
|  |  | Female | -0.04 (0.503) | -0.02 (0.746) | -0.06 (0.537) | 0.1 (0.146) | 0 (0.81) | 0.06 (0.696) |
|  |  | **Age group** | **-0.770** | **-0.010** | **-0.420** | **0.050** | **0.010** | **1.070** |
|  |  | 15-19 | 1.74 (0.001) | -0.09 (0.775) | 1.34 (0.024) | 0 (0.983) | 0.07 (0.233) | 0.46 (0.419) |
|  |  | 20-29 | -0.75 (0.001) | -0.06 (0.734) | -0.21 (0.675) | 0.22 (0.177) | -0.02 (0.25) | 1.74 (0) |
|  |  | 30-39 | -0.68 (0.001) | 0.06 (0.756) | -0.95 (0.007) | 0.04 (0.676) | -0.02 (0.237) | -0.3 (0.172) |
|  |  | 40 + | -1.08 (0.002) | 0.08 (0.743) | -0.6 (0.033) | -0.21 (0.078) | -0.02 (0.235) | -0.83 (0) |
|  |  | **Education level** | 0.260 | -0.020 | 0.260 | 0.170 | 0.010 | 0.400 |
|  |  | None | -0.03 (0.113) | 0.11 (0.734) | 0 (0.977) | -0.04 (0.547) | 0 (0.469) | -0.01 (0.605) |
|  |  | Primary | 0.09 (0.554) | -0.17 (0.724) | 0.44 (0.367) | 0.22 (0.262) | 0 (0.739) | 0.18 (0.547) |
|  |  | Secondary and above | 0.2 (0.142) | 0.04 (0.763) | -0.18 (0.537) | -0.01 (0.773) | 0.01 (0.464) | 0.23 (0.778) |
|  |  | **Standard of living** | **0.220** | **-0.020** | **0.280** | **-0.070** | **0.000** | **0.020** |
|  |  | 1^st^ Quintile | 0.41 (0.047) | -0.02 (0.872) | 0.55 (0.062) | -0.06 (0.635) | 0.01 (0.29) | -0.03 (0.857) |
|  |  | 2^nd^ Quintile | -0.02 (0.368) | 0.01 (0.947) | 0.04 (0.9) | 0.03 (0.676) | 0 (0.613) | 0.09 (0.666) |
|  |  | 3^rd^ Quintile | -0.05 (0.596) | -0.02 (0.844) | -0.08 (0.786) | -0.04 (0.646) | 0 (0.725) | -0.08 (0.646) |
|  |  | 4^th^ Quintile | 0.02 (0.706) | 0 (0.974) | -0.09 (0.203) | 0.06 (0.432) | 0 (0.62) | 0.29 (0.194) |
|  |  | 5^th^ Quintile | -0.14 (0.096) | 0.01 (0.819) | -0.14 (0.611) | -0.06 (0.463) | -0.01 (0.28) | -0.25 (0.365) |
|  |  | **Occupation type** | **1.990** | **-0.020** | **-0.850** | **-0.450** | **-0.050** | **0.040** |
|  |  | Unemployed | 0.46 (0.007) | -0.08 (0.729) | -0.83 (0.035) | -0.25 (0.2) | -0.02 (0.24) | -0.26 (0.464) |
|  |  | Agricultural | 0.31 (0.007) | 0 (1) | -0.1 (0.866) | -0.17 (0.322) | -0.01 (0.237) | 0.24 (0.422) |
|  |  | Domestic | -0.02 (0.001) | -- | 0.05 (0.451) | -- | -- | 0.05 (0.55) |
|  |  | Trade | 0 (0.307) | 0.01 (0.77) | -0.11 (0.398) | -0.01 (0.509) | 0 (0.685) | -0.02 (0.752) |
|  |  | Manual labor | 1.24 (0.008) | 0.04 (0.723) | 0.02 (0.924) | -0.06 (0.373) | -0.02 (0.213) | 0.17 (0.515) |
|  |  | Office / service | -0.05 (0.66) | 0 (0.723) | 0.15 (0.179) | 0.01 (0.336) | 0.01 (0.231) | -0.2 (0.124) |
|  |  | Professional / manager | 0.05 (0.04) | 0.01 (0.753) | -0.03 (0.749) | 0.03 (0.186) | -0.01 (0.267) | 0.06 (0.539) |
|  |  | **Marital status** | **-0.440** | **0.140** | **-1.260** | **-0.200** | **0.060** | **1.020** |
|  |  | Never married | 0.2 (0.035) | -0.06 (0.786) | 1.13 (0.094) | -0.13 (0.566) | 0.12 (0.233) | 2.43 (0.003) |
|  |  | Married | -0.68 (0.013) | 0.2 (0.74) | -2.39 (0.026) | -0.08 (0.675) | -0.06 (0.24) | -1.3 (0.008) |
|  |  | Separated / divorced / widowed | 0.04 (0.301) | 0 (0.78) | 0 (0.989) | 0.01 (0.294) | 0 (0.232) | -0.11 (0.033) |
|  | **Sexual Behavior Factors** | | **0.610** | **0.310** | **1.280** | **-0.370** | **0.010** | **-0.790** |
|  |  | **sexual behavior risk** | **0.240** | **-0.180** | **-0.070** | **-0.180** | **-0.010** | **-0.260** |
|  |  | No | 0.26 (0.276) | -0.18 (0.78) | -0.08 (0.958) | -0.19 (0.728) | -0.01 (0.741) | -0.28 (0.775) |
|  |  | Yes | -0.02 (0.276) | 0 (0.78) | 0.01 (0.958) | 0.01 (0.728) | 0 (0.741) | 0.02 (0.775) |
|  |  | **Premarital sex** | **-0.090** | **0.020** | **0.480** | **0.020** | **0.000** | **0.190** |
|  |  | No | 0.07 (0.375) | 0.03 (0.868) | -0.38 (0.113) | 0.19 (0.148) | 0 (0.648) | -0.47 (0.072) |
|  |  | Yes | -0.16 (0.375) | -0.01 (0.868) | 0.86 (0.113) | -0.17 (0.148) | 0 (0.648) | 0.66 (0.072) |
|  |  | **Multiple sex partners** | **-0.010** | **0.000** | **0.210** | **-0.210** | **0.000** | **-0.660** |
|  |  | No | 0.09 (0.338) | 0 (0.991) | 0.28 (0.72) | -0.23 (0.535) | 0 (0.979) | -0.92 (0.147) |
|  |  | Yes | -0.1 (0.338) | 0 (0.991) | -0.07 (0.72) | 0.02 (0.535) | 0 (0.979) | 0.26 (0.147) |
|  |  | **Age at first sex** | **0.470** | **0.470** | **0.660** | **0.000** | **0.020** | **-0.060** |
|  |  | Never had sex | -0.22 (0.007) | 0.59 (0.717) | -0.5 (0.276) | 0.08 (0.774) | 0.07 (0.243) | -0.93 (0.21) |
|  |  | < 16 years | 0.23 (0.059) | -0.05 (0.715) | 0.62 (0.123) | 0.07 (0.365) | -0.01 (0.26) | 0.08 (0.545) |
|  |  | 16-17 | 0.09 (0.399) | -0.02 (0.733) | 0.14 (0.607) | -0.04 (0.345) | -0.01 (0.257) | -0.01 (0.974) |
|  |  | 18-19 | 0.27 (0.002) | -0.06 (0.73) | -0.05 (0.873) | -0.01 (0.875) | -0.01 (0.292) | 0.09 (0.651) |
|  |  | 20 + | 0.1 (0.564) | 0.01 (0.95) | 0.45 (0.242) | -0.1 (0.42) | -0.02 (0.251) | 0.71 (0.05) |
|  | **HIV ⁄AIDS Awareness** | | **0.160** | **0.060** | **0.010** | **0.010** | **0.000** | **-0.010** |
|  |  | **HIV ⁄AIDS awareness** | **0.160** | **0.060** | **0.010** | **0.010** | **0.000** | **-0.010** |
|  |  | Low | 0.12 (0.386) | -0.09 (0.74) | 0.24 (0.386) | -0.02 (0.776) | 0 (0.953) | -0.31 (0.189) |
|  |  | Average | 0.12 (0.285) | -0.05 (0.747) | -0.03 (0.896) | 0.04 (0.691) | 0.01 (0.366) | 0.2 (0.362) |
|  |  | High | -0.08 (0.055) | 0.2 (0.726) | -0.2 (0.408) | -0.01 (0.94) | -0.01 (0.345) | 0.1 (0.635) |
| **Constant** | | | **1.22 (0.037)** | **-0.77 (0.745)** | **5.4 (0.03)** | **2.29 (0.014)** | **0.35 (0.239)** | **2.75 (0.064)** |
| ^a^: Domestic category not collected  ^b^ Represent the contribution to gender inequalities in HIV/AIDS prevalence due to gender differences in the distributions of observable HIV/AIDS risk factors between women and men  ^c^ Reflect the contribution to gender inequalities in HIV/AIDS due to gender differences in the effects of measured HIV/AIDS risk factors, as well as unmeasured factors not included in the model | | | | | | | | |
